# Quantification of low-temperature gas emissions reveals CO_2_ flux underestimates at Soufrière Hills volcano, Montserrat

**DOI:** 10.1126/sciadv.ads8864

**Published:** 2025-04-02

**Authors:** Alexander Riddell, Mike Burton, Ben Esse, Brendan McCormick Kilbride, Antonio Chiarugi, Thomas Christopher, Francesco D’Amato, Graham A. Ryan, Adam Stinton, Silvia Viciani

**Affiliations:** ^1^ School of Earth and Environmental Science, University of Manchester, Manchester, UK.; ^2^ SENSIT Technologies EMEA Srl, Bolzano, Italy.; ^3^ Montserrat Volcano Observatory, Montserrat, West Indies.; ^4^ Seismic Research Centre, The University of the West Indies, St. Augustine, Trinidad & Tobago.; ^5^ CNR–National Institute of Optics, Firenze, Italy.

## Abstract

We performed helicopter-borne optical MultiGAS measurements of volcanic gas emissions from Soufrière Hills Volcano, Montserrat, revealing distinct spikes in SO_2_ and HCl concentrations within a larger CO_2_-rich plume. Acid-rich concentration spikes matched the distribution of high-temperature fumaroles, whereas CO_2_ is emitted broadly from high- and low-temperature fumaroles. The CO_2_ flux was 15 to 41 kilograms per second from hot fumaroles and 61 to 131 kilograms per second for the overall plume. The typical CO_2_ flux calculation of multiplying CO_2_/SO_2_ ratio with SO_2_ flux underestimates total CO_2_ flux by at least threefold. We quantified substantial magmatic gas scrubbing by the hydrothermal system, with 56 to 79% of initial HCl and 33 to 68% of initial SO_2_ lost. This study highlights the importance of precise acid-gas measurements for detecting heterogeneous degassing and suggests that traditional CO_2_ flux measurements may substantially underestimate contributions from cold CO_2_ degassing, leading to underestimated global volcanic fluxes.

## Teaser

Volcanic gas measurements at Soufrière Hills reveals cold CO_2_ emissions dominate volcanic flux, challenging established methods.

## INTRODUCTION

Globally, over 800 million people live within 100 km of an active volcano (*1*). It is therefore important to monitor volcanoes to detect eruption precursors (*2*) and track ongoing eruptions (*3*, *4*). Gas flux and composition monitoring are a critical tool used by volcano observatories (*5*), providing key insights into the magma dynamics driving volcanic activity (*6*, *7*). Monitoring of gas chemistry and flux in near real time has played a key role in numerous eruption crisis response scenarios, for example, Pinatubo 1991 (*8*), Montserrat 1995 to 2010 (*9*), Etna 2002–2003 (*10*), Stromboli 2002 (*11*) and 2007 (*12*), and Kilauea 2018 (*13*). Accurately measuring the total volatile flux from volcanoes is essential to quantify global volatile cycles (*14*, *15*) and understand the evolution of Earth’s atmosphere and climate (*16*, *17*).

SO_2_ emission rate is commonly used to monitor volcanic unrest, owing to the low background concentration of SO_2_ in the atmosphere and strong absorption spectra in both ultraviolet (UV) and infrared (IR) wavelengths (*18*). SO_2_ emission rate can be combined with compositional analysis of volcanic plumes to calculate the emission rate of other volcanic volatiles, such as CO_2_, H_2_O, HCl, and HF (*19*). That is, by multiplying the SO_2_ emission rate with the X/SO_2_ mass ratio, we can compute the emission rate of species X.

In situ, portable multicomponent gas analyzers, such as MultiGAS (*20*, *21*), are routinely used for measuring the relative abundance of major gas components of volcanic plumes. This is due, in part, to their relatively low cost as they use a combination of off-the-shelf electrochemical and IR sensors for SO_2_ and CO_2_, respectively. Their portability and telemetry capabilities allow for deployment in hazardous volcanic craters, preventing repeat visits for sample collection and costly laboratory analyses.

Volcanic systems are, however, intrinsically complex, and so, untangling the history of these gases as they rise from depth poses many challenges. Gas compositions at open-vent volcanoes may vary due to changing contributions of volatiles exsolved from magma over a range of depths (*22*, *23*). Magmatic degassing can also be modulated by shallow hydrothermal systems (*24*) or crater lakes (*25*), as well as by hydrolysis and scrubbing reactions occurring at temperatures below 400°C (*26*), which modifies the original magma-derived gas composition. The degree of scrubbing will depend on the solubility of each gas. CO_2_ and H_2_S are less soluble than SO_2_ and the halogens and so pass more freely through the hydrothermal system. At volcanoes with well-established hydrothermal systems, monitoring strategies must recognize the potential for the removal of acidic gases by scrubbing, either through comparisons with fumarole temperatures or monitoring of less soluble species such as CO_2_ and H_2_S (*27*). Without careful consideration of scrubbing, gas relative abundance ratios and absolute emission rates may not accurately reflect the present state of the magmatic system, limiting the capability of these data for monitoring the state of activity, precursor detection, and eruption forecasting.

Soufrière Hills Volcano (SHV), Montserrat, is an open-vent volcano that exhibits steady-state magmatic degassing via a shallow hydrothermal system (*28*). The volcano is situated within the Caribbean arc, where magmatism is driven by the subduction of the North American plate beneath the Caribbean plate, with the slab lying at 120 km depth beneath Montserrat (*29*). The episodic eruption at Soufrière Hills between 1995 and 2010 consisted of five phases of andesitic lava dome extrusions and collapses (*30*, *31*), cumulatively producing over a cubic kilometer of lava (*31*). A deep underplating of mafic magma beneath a cooler andesite is widely accepted as the mechanism driving eruptible magma production, as evidenced by the presence of mafic enclaves in the erupted andesite (*32*). Analysis of phase equilibrium and preeruptive melt contents has estimated the depth to the top of the magma chamber to be 5 to 7 km deep (*33*), which agrees also with geophysical observations (*34*).

The Montserrat Volcano Observatory (MVO) maintains and operates several volcanic monitoring systems, including seismic stations. Soufrière Hills has been continuously emitting SO_2_ since the start of the 1995 eruption (*35*). Continuous monitoring of SO_2_ gas emissions was established in 2002 using a network of scanning UV spectrometers (*36*), but since 2017, most monitoring of SO_2_ flux has depended on manually performed traverse measurements. During the measurement period described here, the MVO reported a SO₂ flux of 140 to 400 tons/day, which is consistent with the range observed during previous pause periods (*37*). Like many arc volcanoes, the total mass of sulfur released over the eruptive cycles is often higher than can be accounted for by the erupting andesite alone (*38*), commonly referred to as excess sulfur (*39*, *40*). At Soufrière Hills, the excess sulfur is sourced by a sulfur-rich vapor phase originating from the intruding mafic magma (*9*). HCl fluxes increase due to shallow degassing during eruptions (*41*), partitioning from ascending andesitic magma into the coexisting fluid phase during decompression (*38*), but is also released between eruptive periods (*42*), indicating a deep source of HCl. Variations in the HCl surface emission may be further modulated by intermittent sealing of the conduit by precipitating hydrothermal fluids (*28*); however, the SO₂/HCl ratio appears to have remained steady for many years, suggesting a limited role for precipitation-driven modulation in degassing at SHV.

**Fig. 1.**
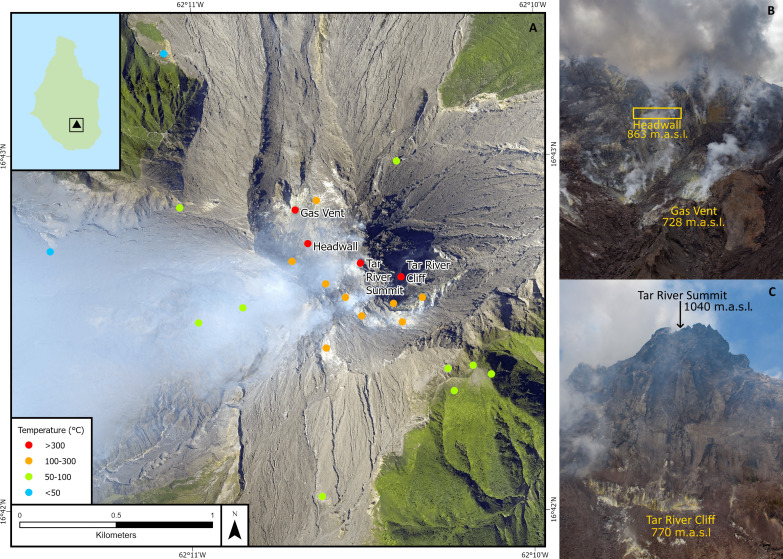
Map of the fumarole fields at SHV. (**A**) Map of the summit of the SHV lava dome showing the location of the four hottest fumaroles (red dots) surrounded by other fumaroles of varying temperatures on and proximal to the lava dome. The black box in the insert shows the position of the main figure. (**B**) Photograph showing the relative positions of the Gas Vent and Headwall fumaroles inside the 2010 collapse scar on the northern flank of the lava dome. m.a.s.l., m above sea level. View looking south (**C**) Photograph showing the relative positions of the Tar River Cliff and Tar River Summit fumaroles on the eastern flank of the lava dome. View looking west. Photos by A.S., MVO.

The summit of SHV is characterized by multiple fumaroles with temperatures ranging from less than 100°C to more than 500°C (Fig. 1). Fumarole temperatures are monitored by a FLIR camera during helicopter observation flights. In the month before this study (April 2017), there were four hot gas-emitting fumaroles: Gas Vent (359°C), Headwall (465°C), Tar River Cliff (321°C), and Tar River Summit (297°C). These four hottest fumaroles are encircled by progressively cooling fumaroles with gas emission replaced by steam at lower temperatures and eventually heated ground fumaroles (Fig. 1). It is from these four hottest fumaroles that we can expect the least amount of scrubbing to occur and so be the dominant source of the more soluble acidic gas emission.

With the continued deformation (*34*), seismicity, and volcanic emissions (*9*) at Soufrière Hills, in the absence of eruptive activity since 2010, any previously unidentified insights into the magma feeding system and degassing processes are extremely valuable in assessing the ongoing risks posed by the volcanic system to the people of Montserrat.

In this work, we report results on SHV plume composition measurements using helicopter-borne in situ tunable diode laser and light-emitting diode absorption spectroscopy—an optical MultiGas (OMG). This instrument uses open-path multipass optical cells to quantify CO_2_, SO_2_, HCl, and HF concentrations at 2- to 2.5-Hz frequency, overcoming some of the limitations of normal MultiGAS systems, thanks to fast response times and utilization of purely optical sensors, allowing traceable and reproducible data. The total weight of the instrument is ~20 kg, so it remains quite portable. OMG measurements were acquired ~2 km downwind from the summit together with airborne traverse measurements of SO_2_ column amounts acquired using a UV spectrometer. This allowed quantification of SO_2_ flux and calculation of the emission rates of CO_2_ and HCl; however, HF concentrations were below the detection limit [25 parts per billion (ppb)] of the OMG. We detect distinct hot plumes generated from high-temperature fumaroles, mixed with wider lower temperature emissions surrounding the lava dome. These hot plumes are well matched spatially and persistently with the distribution of hot fumaroles in the crater. We measure an overall CO_2_ flux at least 3.5x higher than previously reported by Edmonds *et al.* (*43*), thanks to the increased sensitivity and frequency of the OMG allowing for quantification of cold volcanic CO_2_ emissions.

## RESULTS

### Sulfur dioxide emissions

**Fig. 2.**
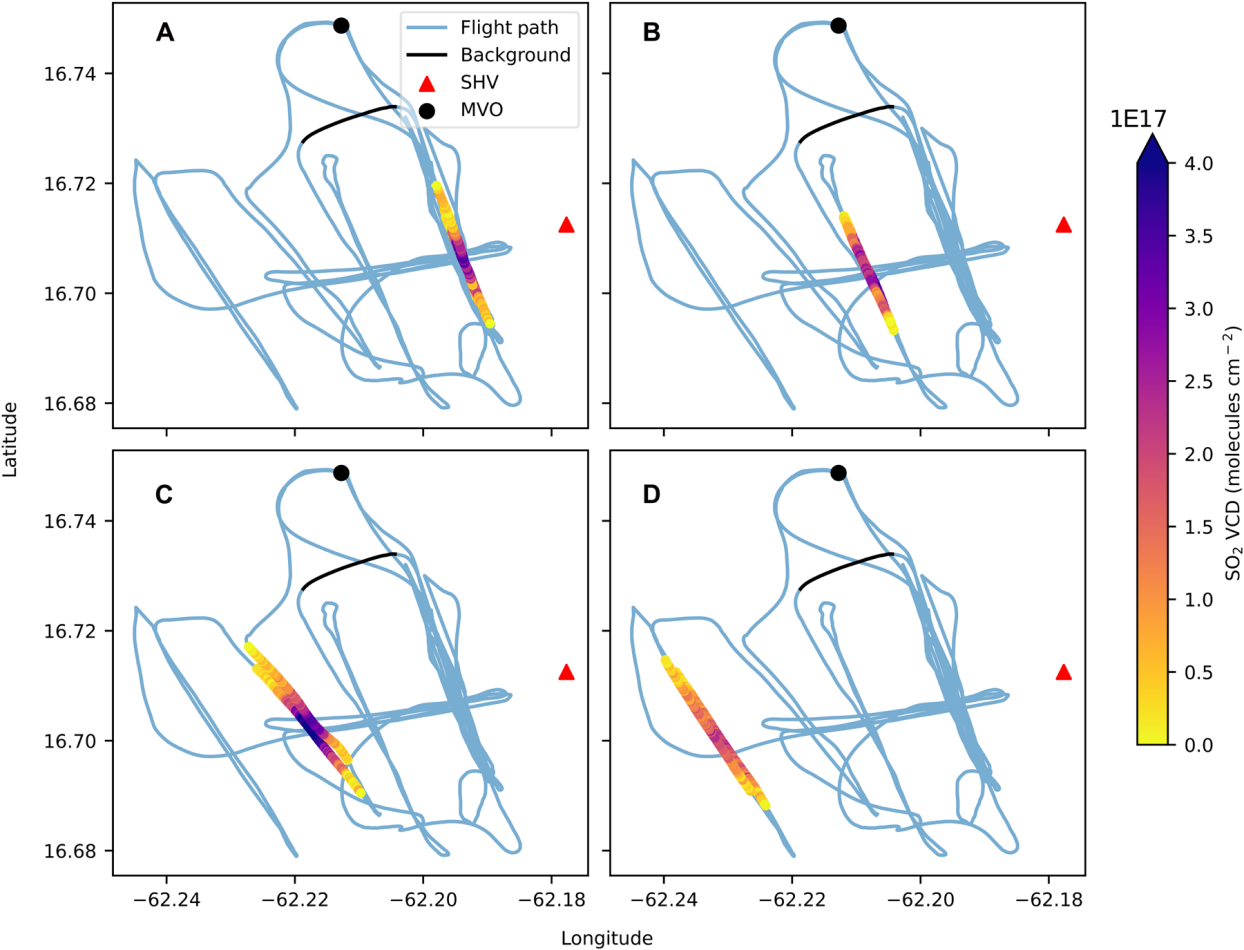
Flight path showing the SO_2_ VCD amount for each of the four different traverse locations. (**A**) 2 km, (**B**) 3.5 km, (**C**) 4.5 km, (**D**) and 6 km downwind of the summit. The black line represents the section of the flight path used to determine the background concentrations of gas, outside of the volcanic plume.

SO_2_ emission measurements were conducted on 19 May 2017 with 10 under-plume traverses at distances of 2, 3.5, 4.5, and 6 km from the summit of SHV, using an Ocean Optics USB2000 UV spectrometer. Figure 2 shows the vertical column density (VCD) of SO_2_ retrieved with iFit (*44*) along the helicopter flight path. The resulting emission rates for each traverse were calculated using GPS locations and measured wind speeds, producing an average emission of 4.03 ± 0.9 kg s^−1^ (table S1).

### Gas composition

In-plume gas composition data were measured using the OMG. To improve sensitivity to CO_2_, the laser did not scan across H_2_O absorption lines, so H_2_O was not measured. To define the start and end of the plume for each traverse, we established a set of requirements: A minimum of 20 data points must be present, with no more than three consecutive values falling below the background concentration. In addition, the peak amplitude must exceed a value three times greater than the SD above the background concentration. The results from these selection criteria are then verified to make sure that they are located appropriately along our traverse, i.e., not selected when the helicopter is turning around.

**Fig. 3.**
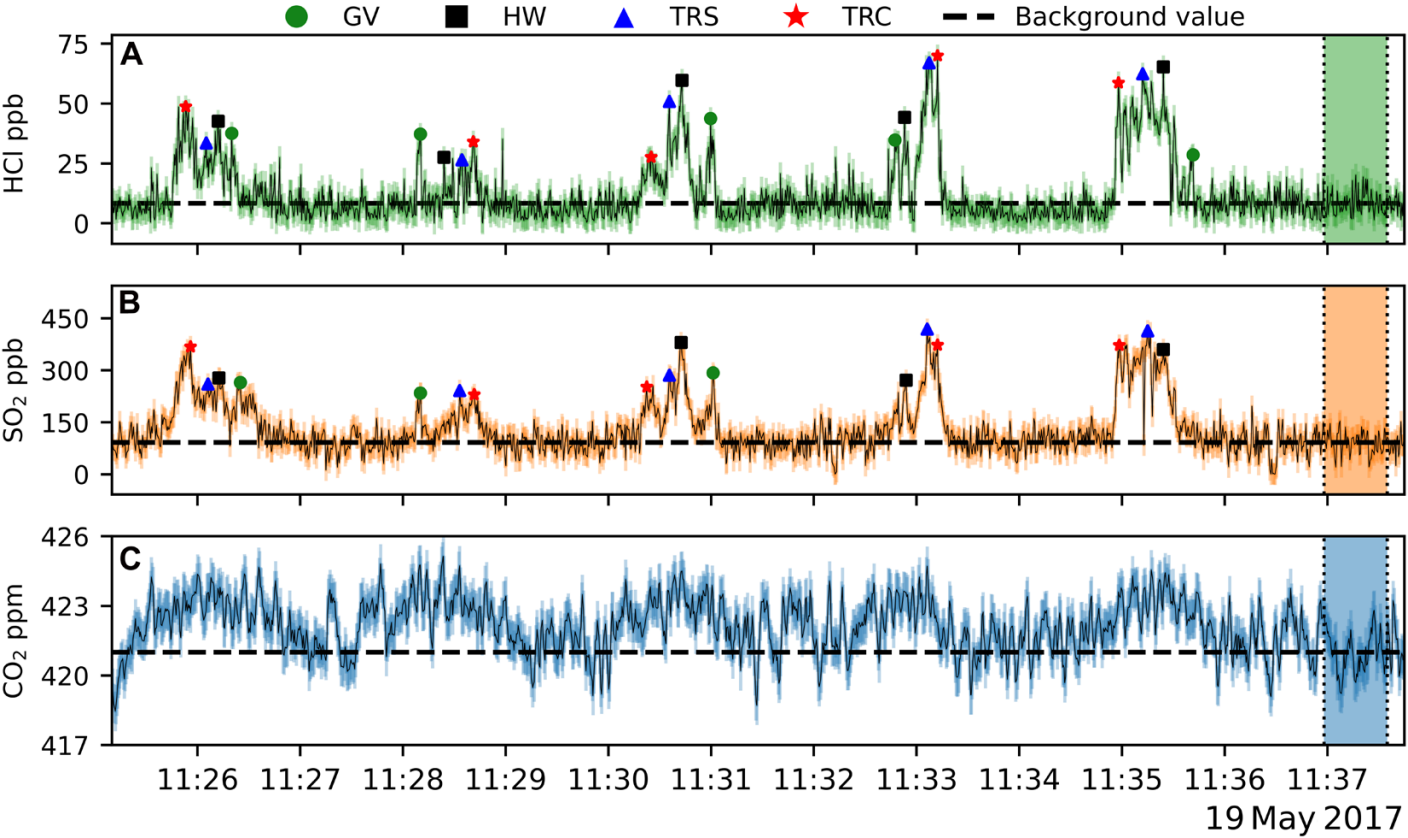
Time series of OMG measurements at SHV. Individual gas concentrations are shown by; (**A**) HCl, (**B**) SO_2_, and (**C**) CO_2_. Horizontal dashed lines represent the average value of each gas measured in the area outside of the plume, marked by the vertically dotted and shaded region for each gas species. Errors (±1σ) are calculated empirically from this window and shown in the shaded colors around the concentration time series in black. The preserved peaks of individual fumaroles are represented by the markers on the SO_2_ and HCl time series as discussed in OMG plume structure (GV, Gas Vent; HW, Headwall; TRC, Tar River Cliff; TRS, Tar River Summit).

Figure 3 presents a time series of CO_2_, SO_2_, and HCl concentrations during the helicopter flight on 19 May 2017. Volcanic CO_2_ was measured up to 4 parts per million (ppm) above background, SO_2_ concentrations were up to 450 ppb, and HCl was up to 70 ppb, with a typical noise of 0.85 ppm, 31 ppb, and 4.7 ppb, respectively. Background concentrations for CO_2_, SO_2_, and HCl were measured 1.7 km from the plume peak concentration location (see Fig. 2) comprising 75 data points, highlighted by the vertical dotted lines in Fig. 3. The following five traverses were conducted with the OMG 2 km downwind of the summit at an altitude between 330 and 360 m above sea level and are referred to as traverses 1 to 5.

We calculate the gas mixing ratios using the measurements from the OMG using a variety of different methods (see Gas composition calculations). The molar ratios are presented in Table 1.

**Table 1.** Calculated molar ratios based on OMG data. The plume is split into the central unscrubbed plume defined by the SO_2_ plume width and a second overall ratio that encompasses all emissions from Soufrière Hills.

Gas source	CO_2_/SO_2_ (molar)	CO_2_/HCl (molar)	HCl/SO_2_ (molar)
Central plume (point-to-point)	10 ± 4	59 ± 26	
Central plume (regression line)			0.20 ± 0.01
Overall SHV (integration)	22 ± 14	166 ± 96	
Overall SHV (optimized geometry)	25 ± 15	183 ± 101	0.14 ± 0.05
Overall SHV (circular geometry)	42 ± 25	470 ± 261	0.09 ± 0.03

### Heterogeneous plume

**Fig. 4.**
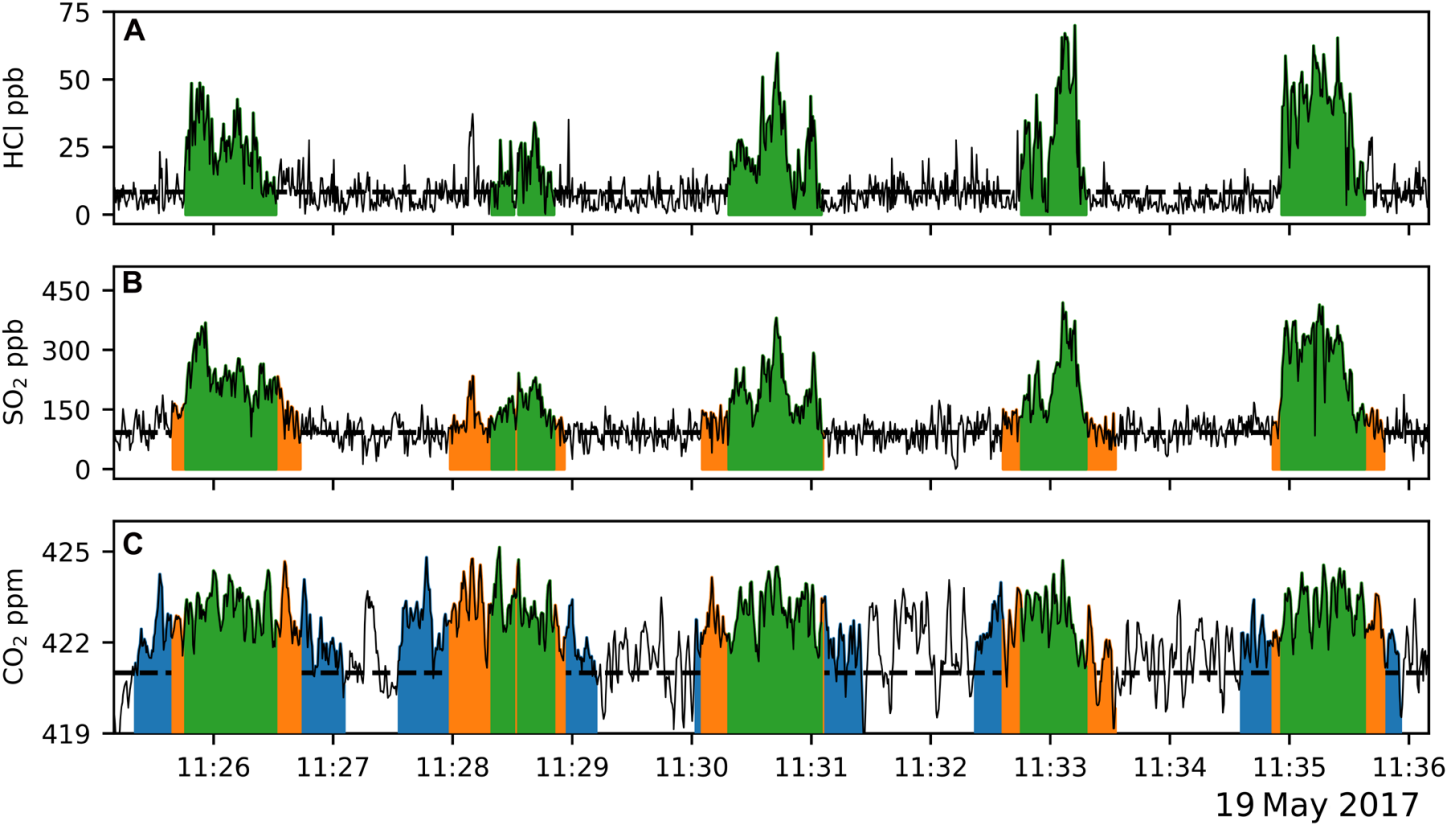
Different plume widths overlaid on each gas species. The width of each gas plume, as determined using the selection criteria, are highlighted in green, orange, and blue for (**A**) HCl, (**B**) SO_2_ and (**C**) CO_2_, respectively.

The high sampling frequency of the OMG reveals a spatially heterogeneous gas composition with an average CO_2_ plume width of 2350 m, SO_2_ plume width of 1494 m, and HCl plume width of 1010 m, with the latter two centrally located within the wider CO_2_ plume (Fig. 4). We propose that this is a feature of the hydrothermal system on SHV, where more soluble SO_2_ and HCl are scrubbed in cooler fumarole emissions, whereas the less soluble CO_2_ is emitted freely. We therefore produce a gas mixing ratio for the acidic central plume and a second for the entire volcanic output, including the wider CO_2_ emission. The SO_2_ and HCl measurements from traverse two are much lower than those of the other traverses, most likely due to intercepting the plume too low and so are excluded from the calculation of gas mixing ratios but are included in Fig. 4 to demonstrate the plume identification procedure.

### Gas fluxes

The emission rate of CO_2_ and HCl for both the central plume and the overall plume are calculated by multiplying the mass ratios of (CO_2_/HCl)/SO_2_ with the SO_2_ flux from the UV spectrometer. The fluxes from each gas for the different sources are reported in Table 2.

**Table 2.** Volcanic gas emission rate at Soufrière Hills. SO_2_, CO_2_, and HCl emission in kilograms per second from the central, hotter fumaroles and the entire volcanic emissions.

Gas	Central plume (kg s^−1^)	Overall SHV plumeintegration (kg s^−1^)	Overall SHV plumeoptimized geometry (kg s^−1^)	Overall SHV plume circular geometry (kg s^−1^)
SO_2_	4.0 ± 0.9		4.0 ± 1.2	4.5 ± 1.4
CO_2_	28.1 ± 13.1	61.3 ± 39.9	68.3 ± 34.8	130.6 ± 66.0
HCl	0.46 ± 0.11		0.31 ± 0.07	0.23 ± 0.05

## DISCUSSION

### Scrubbing

The geothermal potential of Montserrat was investigated prior to the 1995 eruption due to the presence of many hot springs and fumarole fields across the island (*45*). ^3^He/^4^He ratios of 5.9 to 8 R_A_ were measured across the fumaroles on Soufrière Hills by Chiodini *et al.* (*45*), which are typical of high-temperature volcanic arc emissions globally (*14*) and thus provide evidence for a magmatic source of the fluid.

The detection of a heterogeneous plume at Soufrière Hills can be explained due to the scrubbing of the water-soluble sulfur dioxide and hydrogen chloride by the hydrothermal system (*26*). The CO_2_ is less likely to undergo such processes owing to its lower solubility and preference for the gaseous phase during boiling. At temperatures less than 400°C, the hydrolysis of SO_2_ is increasingly favored by the following reaction (*26*)

$$4{\text{SO}}_{2\left(g\right)}+4H_{2}O_{\left(\text{aq}\right)}\to H_{2}S_{\left(\text{aq}\right)}+3H_{2}SO_{4\left(\text{aq}\right)}$$ (1)

and

$$3{\text{SO}}_{2\left(g\right)}+2H_{2}O_{\left(\text{aq}\right)}\to S_{\left(s,l\right)}+2H_{2}SO_{4\left(\text{aq}\right)}$$ (2)

HCl is removed from the gas phase by the following reaction (*26*)

$${\text{HCl}}_{\left(g\right)}\to H_{\left(\text{aq}\right)}^{+}+Cl_{\left(\text{aq}\right)}^{-}$$ (3)

Comparison of the SO_2_ and HCl plumes shows very similar shapes and spatial alignment of peaks in the datasets over the width of the HCl plume, indicating that they are from the same source (Fig. 4). HCl concentrations drop sharply to background levels, suggesting that these emissions are solely from the four hottest fumaroles, likely due to HCl’s high water solubility. The SO_2_ plume shows a more diffuse edge, extending on either side of the HCl gas plume, as shown in Fig. 4. We refer to this portion of the SO_2_ plume as the “SO_2_ edge.” Most SO_2_ is sourced from the four hottest fumaroles; however, the SO_2_ edge is ascribed to minor contributions from cooler fumaroles (<300°C but >100°C) immediately surrounding the hottest fumaroles (Fig. 4 and Fig. 1). The SO_2_ edge measurements align with spikes in the CO_2_ emissions (Fig. 4), which confirms that the SO_2_ edge is from fumaroles that are cooler than the central SO_2_ plume gas. Volcanic CO_2_ measured past the SO_2_ edge is emitted from only the coolest emitting fumaroles, below 100°C, resulting in completed hydrolysis of SO_2_ and HCl.

### Plume composition and flux comparison

**Fig. 5.**
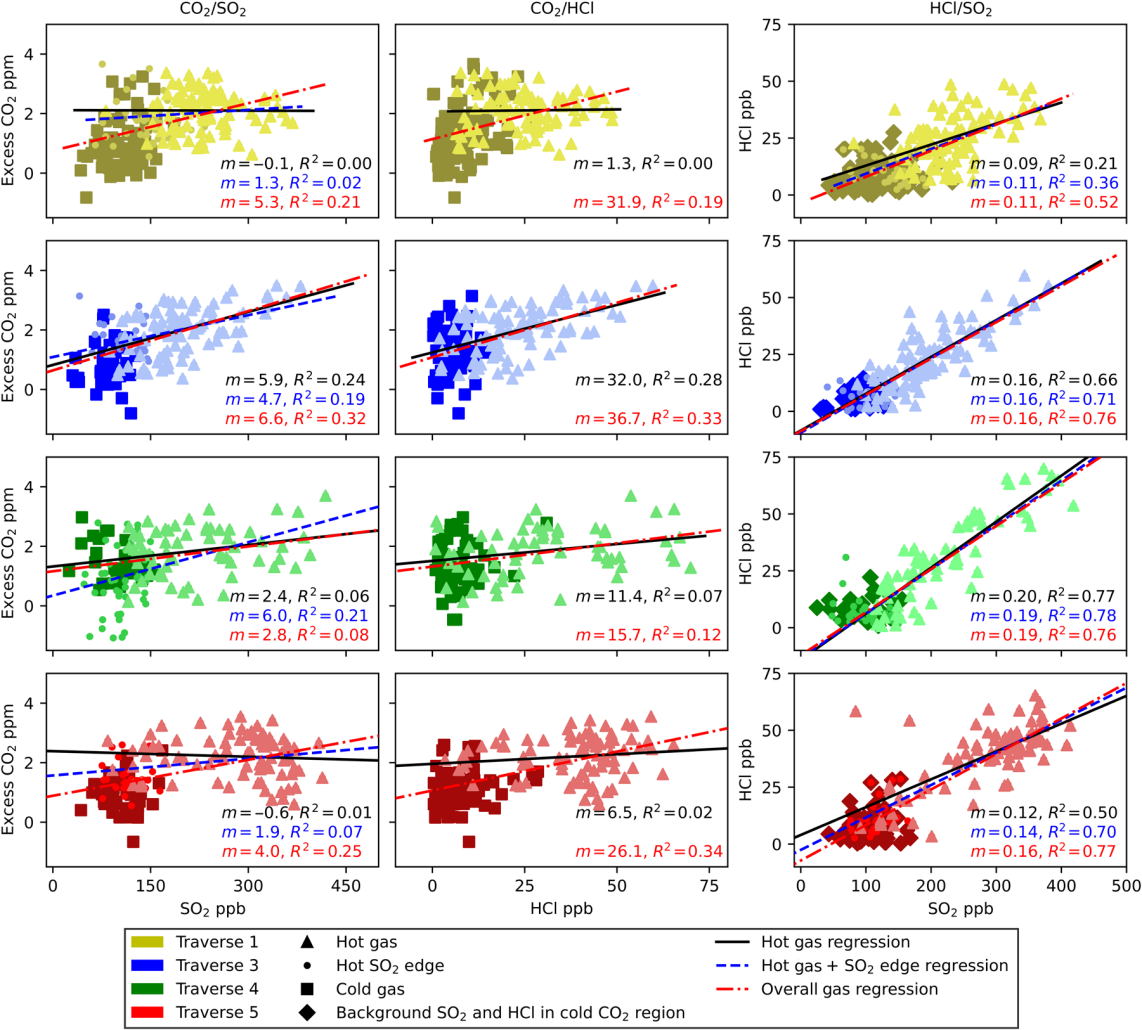
Scatterplots for the different gas species across traverses 1, 3, 4, and 5. The black regression lines represent gas emitted within the width of the HCl plume (hot gas), the blue line represents all measurements within the width of the SO_2_ plume (hot gas including SO_2_ edge), and the solid black lines represent the regression for the overall emission, defined by the width of the CO_2_ plume. Different shades of the traverse color key are used to help differentiate the symbols for each traverse. The regression coefficient and correlation coefficient are shown for the regression line on each subplot, with the text color matching the regression line. The first column lists the CO_2_/SO_2_ ratio, the second column the CO_2_/HCl ratio, and the third column the HCl/SO_2_ ratio. The rows correspond to traverses as follows: The first row represents traverse 1, the second row traverse 3, the third row traverse 4, and the fourth row traverse 5.

Scatterplots of species X versus Y are commonly used to retrieve gas mixing ratios from MultiGAS and Open-Path Fourier Transform Infrared (OP-FTIR) instruments (*20*, *23*, *42*, *46*). Measurements within the plume are selected for each gas, and a linear regression is used to determine the mixing ratio. Applying this approach to the OMG data at Soufrière Hills, we achieve a high correlation between HCl and SO_2_ (Fig. 5); however, due to the high sampling rate and sensitivity to CO_2_, we introduce greater noise into the scatterplots featuring CO_2_. Another problem with plotting the CO_2_/(SO_2_/HCl) is due to the heterogeneity of the plume. The SO_2_ and HCl plumes are not perfectly symmetrical within the CO_2_ plume across all traverses, and so we see poor correlation between species such as in traverses 4 and 5 (Fig. 5). Increasing the degree of smoothing applied to the CO_2_ cannot correct for this asymmetric heterogeneity. When the inner SO_2_ and HCl plumes are symmetrically located with respect to the CO_2_ plume, such as traverse 3, it is possible to derive a mixing ratio. However, we favor the use of the integration method for determining the overall mixing ratios at Soufrière Hills involving CO_2_ as a more robust approach.

The first measurements of the plume at Soufrière Hills using a MultiGAS instrument were achieved in 2008, between the 4th and 5th eruptive phase by Edmonds *et al.* (*43*). In which, they report a molar CO_2_/SO_2_ ratios for Soufrière Hills of 5.1, coupled with a similar SO_2_ flux as reported in this study of 400 tons day^−1^, producing a daily CO_2_ emission of 1468 tons. We conclude that the ratios produced by Edmonds *et al.* (*45*) may be influenced by excluding results where the SO_2_ concentration falls below 1 ppm as this would systematically remove plume compositions from the cooler fumaroles where the SO_2_ is minimal but CO_2_ remains high. This would have the effect of lowering the CO_2_/SO_2_ ratio and the subsequent CO_2_ flux. However, as with other MultiGAS studies, Edmonds *et al.* (*43*) filter the MultiGAS data for gases with such low concentrations due to lower sensitivity and higher noise of the MultiGAS. The MultiGAS instrument was stationary throughout the study by Edmonds *et al.* (*43*), which again may have preferentially sampled only the hot fumarole emissions. Without strong changes in wind direction, the sensors may not have been exposed to the CO_2_-rich, SO_2_-poor cooler fumarole emissions from the wider flanks of the volcano. With the assumption that the gas compositions from Edmonds *et al.* (*43*) are representative of only the hot fumarole emission, we produce a hot gas CO_2_ flux almost 50% higher than in 2008. The higher CO_2_ flux from the summit area in this study may be elevated by the inclusion of colder fumaroles upwind of the hottest fumaroles (Fig. 1).

### HCl flux

Measurements of chlorine emission from Soufrière Hills have been obtained by solar occultation FTIR throughout the eruption [see Christopher *et al.* (*38*) for a full timeline]. Edmonds *et al.* (*47*) and Christopher *et al.* (*38*) conclude that an increase in the HCl flux coincides with periods of higher extrusion rate and that the source must be degassing of the ascending andesite magma during eruptions. During the eruptive phases, the HCl/SO_2_ ratio was used as a proxy for the ascent of andesitic magma following an increase in HCl degassing (*42*, *47*). During periods of high extrusive activity, the molar HCl/SO_2_ ratios exceed 1 and emission rates of HCl range from 350 to 10,000 tons day^−1^ (*42*).

We measure molar HCl/SO_2_ ratios of 0.20 ± 0.01, which is in line with previous measurements recorded during pause periods (*38*) and is consistent with a supply of gas from depth, owing to the continuous emission of SO_2_ throughout the previous 13 years of quiescence. As with the SO_2_, HCl surface emissions are best described by a modified Darcy model of permeability due to the sealing of the conduit by precipitating hydrothermal fluids (*28*). The hotter fumaroles on Soufrière Hills will allow for more efficient movement of the HCl to the surface, but it remains likely that a fraction of the HCl will be removed due to its high reactivity. Periods of effusive activity, when hot lava is in the upper dome, will raise the temperature of the hydrothermal system, which may reduce scrubbing reactions and will directly affect the emission of HCl. The initial increase in temperature may be enough to allow for the evaporation of HCl-rich fluids, further adding to the HCl emissions.

### CO_2_ underestimate

The observation of a CO_2_ flux three times greater than previous estimates has been made possible due to the high frequency and rapid sensor response time of the OMG. Accurately quantifying the CO_2_ emission is vital not only for better understanding the magmatic processes occurring within SHV but also for determining global volcanic emission budgets. Many studies have examined the Earth’s CO_2_ flux from actively degassing volcanoes (*48*–*51*). Most of these studies use satellite-based measurements of SO_2_ and combine with CO_2_/SO_2_ measured using the likes of MultiGAS in crater plumes. However, many volcanoes around the world exhibit hydrothermal systems, affecting sulfur speciation and scrubbing of gas, and so can have a twofold effect on the carbon emission retrieved. First, by affecting the satellite retrieved measurement of SO_2_ due to scrubbing and preference for H_2_S speciation (*26*). Second, as shown here, without adequate sensor response times and high species sensitivity, heterogeneous plumes composed of hot and cold gas may be selectively sampled for only their hot composition by MultiGAS instruments. This combined effect would result in a substantial underestimation of CO_2_ flux for a large proportion of volcanoes worldwide, especially for the persistently low SO_2_ flux volcanoes where scrubbing and heterogeneous plumes are more likely to occur. Allard *et al.* (*52*) showed a similar encompassing the “CO_2_ dome” at Mt. Etna, extending 15 km past the edge of the SO_2_ crater plume. By using the approach of scaling the CO_2_/SO_2_ ratio of the crater plume by the SO_2_ flux, Allard *et al.* (*52*) computed a CO_2_ flux of 29 to 120 kiloton CO_2_ day^−1^ for the crater plume. In our case, this is the same approach we used to calculate the CO_2_ flux for the central (hot) plume source. The overarching dome of CO_2_ on Etna was measured by an airborne profile and the soils was sampled for δ^13^C and R/Ra ratios, which confirmed it was of magmatic origin, with the conclusion that the source of this dome was diffuse soil degassing. We suggest that scrubbed fumarole emissions in and around the summit craters may have contributed to the CO_2_ dome. Allard *et al.* (*52*) calculated the true emission of diffuse CO_2_ to be comparable to that of the output of CO_2_ from the crater plume alone (55 kiloton day^−1^ CO_2_ versus 70 kiloton day^−1^ CO_2_, respectively).

### OMG plume structure

**Fig. 6.**
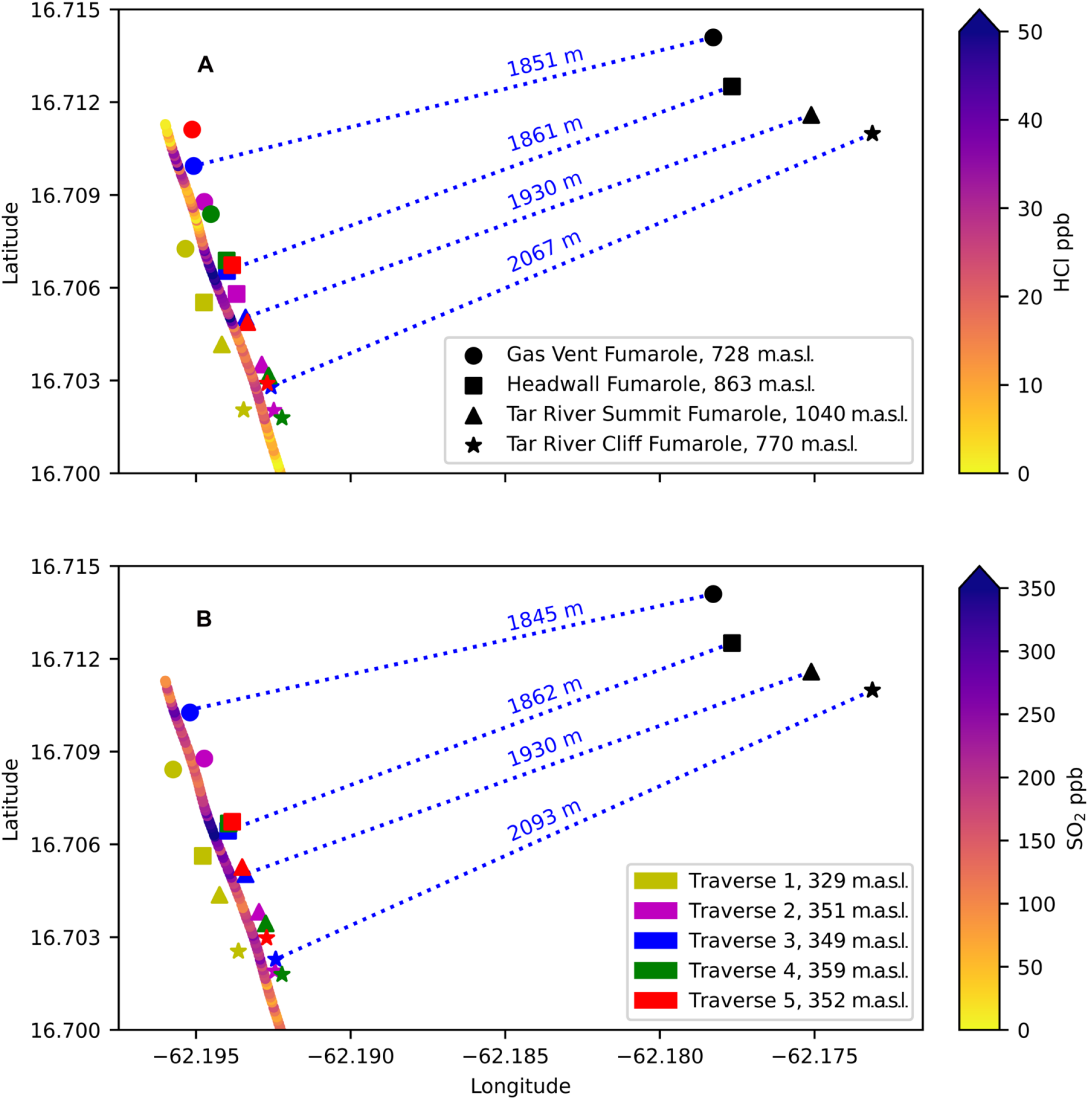
Preservation of individual fumarole contributions downwind from SHV. Plotted location of the peak HCl concentrations (**A**) and SO_2_ concentrations (**B**) across each traverse that corresponds to the individual fumarole source. The legend for the entire figure is split over the two panels. The distance between the fumarole source and the downwind peak concentration are shown by the blue dotted lines for HCl and SO_2_ in (A) and (B), respectively, and are calculated based on traverse 3.

The high frequency and increased sensitivity of the SO_2_ and HCl optical sensors allow us to resolve the individual fumarole contribution to the overall plume, something that has not been achieved by in situ gas sensing methods previously. Across each of the OMG traverses, there are up to four distinct peaks preserved in the SO_2_ and HCl, showing exceptional similarity and alignment between both species (see Fig. 3). By plotting the GPS location corresponding to these peaks, we find that it produces distinct groupings across all traverses and between both SO_2_ and HCl, as presented in Fig. 6 (A and B), respectively. We therefore propose that these peaks preserve the spatial distribution of the four hot fumaroles found on Soufrière Hills.

We highlight that sharp CO_2_ peaks associated with the hot fumaroles are not seen in Fig. 3, providing support for our hypothesis that the CO_2_ plume is abundantly produced from multiple cooler fumarolic sources around the hot fumaroles, thereby dominating the CO_2_ emissions from the higher temperature fumaroles.

**Fig. 7.**
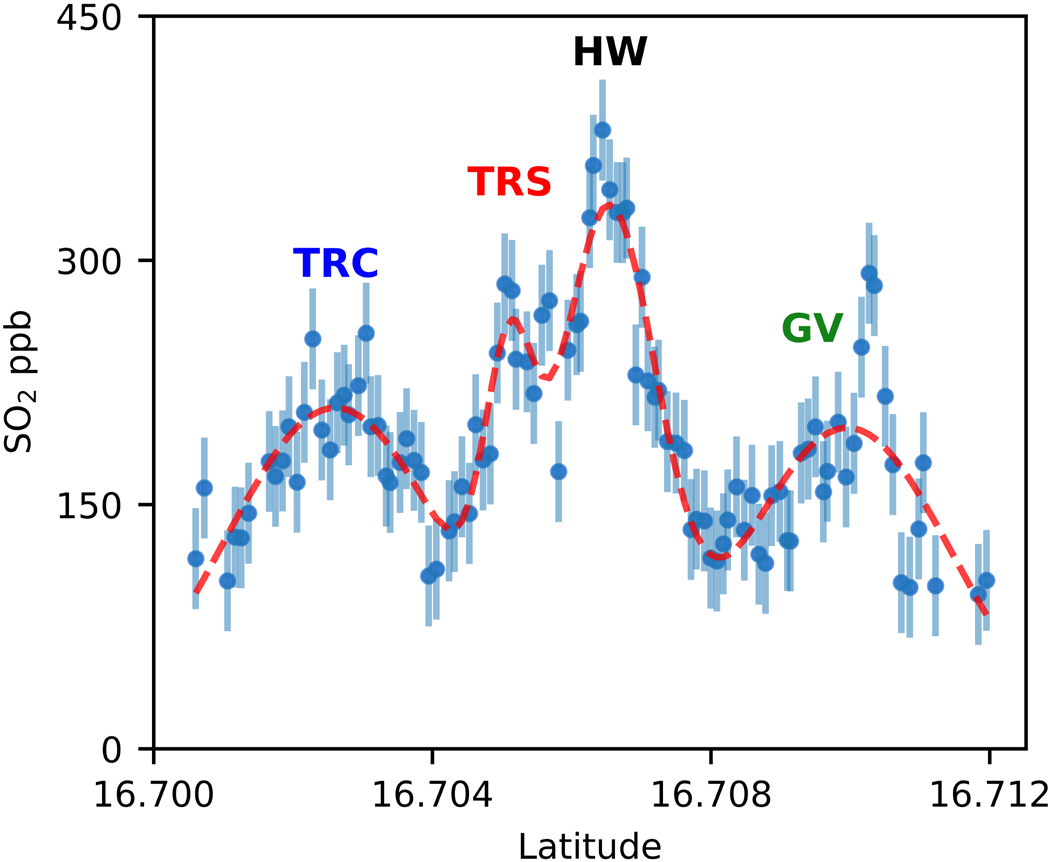
Preserved individual fumarole contribution of SO_2_ in traverse 3. SO_2_ concentrations across traverse 3 are shown by the blue circles, with error bars showing the SD of the background measurements (31 ppb). The four fumaroles’ sources are indicated by letters above the peak: TRS (Tar River Summit), TRC (Tar River Cliff), HW (Headwall), and GV (Gas Vent). The red dashed line represents a function composed of four Gaussian curves fitted to the real SO_2_ data.

The gases originating from the Headwall fumarole appear to be best preserved downwind, with tight spatial grouping and appearing in 9 of 10 of the combined traverses of SO_2_ and HCl. This is expected as it is the hottest fumarole, over 400°C, and so is likely least affected by scrubbing processes and thus more easily detected. The individual fumarole contributions are highlighted when fitting a function comprising four Gaussian curves to the SO_2_ data from traverse 3 (Fig. 7). The measured data can be modeled remarkably well and shows Headwall fumarole masking much of the Tar River Cliff source, although there are some disagreements between the fitted curve and data points for Gas Vent fumarole. In reality, plume shapes do not follow a perfectly Gaussian profile due to constant fluctuations in emission rates and wind shear, so such discrepancies are to be expected. Despite being of higher temperature than Tar River Summit, Gas Vent is captured less across all traverses and shows poorer clustering of peaks. The Gas Vent fumarole is situated on the floor of the 2010 collapse scar and so is subject to highly variable winds that can result in strong mixing within the scar, before being subjected to the wind sheering the plume downwind once it crests the crater rim. This may explain why we see the most lateral variability in the Gas Vent peaks 2 km downwind.

### Unscrubbed emissions

We can estimate the total emission of SO_2_ and HCl in the absence of any hydrothermal interaction by multiplying the CO_2_ flux of the entire Soufrière Hills system by the hot gas CO_2_/SO_2_ and CO_2_/HCl mass ratios, respectively, assuming that this is representative of the gas composition prior to hydrothermal interaction. Table 3 presents the emission rate for SO_2_ and HCl using the three different values for CO_2_ flux. The percentage of each gas scrubbed by the hydrothermal system is reported in relation to the true OMG central (hot) gas measurements. We see that the HCl gas is scrubbed more than SO_2_ across all scenarios as expected due to the higher solubility in water. The calculated error remains high for these values due to the difference between the SD of the background CO_2_ measurement and the amount of excess volcanic CO_2_ measured. Regardless of the CO_2_ flux value used in this calculation, the HCl/SO_2_ molar ratio at depth based on the unscrubbed emission would be 0.14.

**Table 3.** Calculated emission rate of CO_2_ and HCl prior to interaction with the hydrothermal system and scrubbing processes. Calculations are based on the three different values of CO_2_ flux and the CO_2_/(HCl/SO_2_) of the hot gas. The percentage of scrubbing is calculated by the difference between the emission without scrubbing, presented in this table, and the fluxes reported for each method in Table 2.

Overall CO_2_ emission method used	Integration		Optimized geometry		Circular geometry	
Gas species	SO_2_	HCl	SO_2_	HCl	SO_2_	HCl
Emission without scrubbing (kg s^−1^)	6.0 ± 4.3	1.1 ± 0.9	6.7 ± 4.6	1.1 ± 0.9	12.8 ± 8.7	2.1 ± 1.7
Percentage scrubbed %	33	56	39	61	68	79

### What would a standard MultiGAS have seen?

The OMG is especially capable for aerial sampling thanks to its ability for rapid measurement and a wide variety of target molecules compared to traditional MultiGAS instruments, which often struggle to spatially resolve gases at faster air speeds. A comparison of detection techniques for the OMG and MultiGAS instruments can be found in table S2. To evaluate the potential benefits of the OMG’s purely optical array over the mix of sensors used in a MultiGAS instrument, we can pass our data through a series of modeled functions to emulate the MultiGAS sensor characteristics (figs. S1 and S2). We use the electrochemical SO_2_ sensor response effects as reported by Liu *et al.* (*53*). For the SO_2_ sensor, these include resampling the data from 2.5 to 1 Hz, introducing a 3-s delay, a first-order response with a time constant of 3.5 s and applying a normally distributed SD of 93 ppb [as calculated from Liu *et al.* (*53*), Data set 1]. Last, the data are processed within the Ratiocalc software for volcanic gas concentration time series (*54*), where a Savitzky-Golay low-pass least-squares filter is applied with a window length of 13 and polynomial of 2, as per Liu *et al.* (*53*) to reduce noise.

Liu *et al.* (*53*) also report the response of the CO_2_ nondispersive infrared (NDIR) sensor within the MultiGAS. They report a rate-limited response of 35 ppm/s and a delay of 5 s. We reprocess our dataset using the 1-Hz sampling rate and 5-s delay (fig. S2); however, our data do not fluctuate more than 35 ppm over the entire time series. Last, we apply a normally distributed SD of 3.6 ppm [as calculated by Liu *et al.* (*53*), Data set 1]. McCormick Kilbride *et al.* (*23*) account for differing sensor response characteristics of the CO_2_ NDIR by applying a boxcar moving average to the original signal of 15 s, as determined in a laboratory setting by Wood *et al.* (*55*). This model for CO_2_ is representative of the MultiGAS setup used by Liu *et al.* (*53*), Liu and Aiuppa (*56*), Pering *et al.* (*57*), and McCormick Kilbride *et al.* (*23*). However, this model does not account for the effects of pumping the air through the instrument as used in these studies and may further affect the gas mixing and signal delay.

The most notable effect of both datasets is the addition of the larger SD and so the increased noise of the reprocessed data as shown in fig. S2 for SO_2_ and fig. S2 for CO_2_. The Savitzky-Golay filter effectively smooths the noisy SO_2_ data but reduces the amplitude of the plumes and loses the fine structures preserving the individual fumarole contributions. The delay of the electrochemical SO_2_ sensor can introduce substantial location errors, with a 3-s delay at a traverse speed of 30 ms^−1^ equal to a 90-m lateral offset. The resulting CO_2_ flux, using the CO_2_/SO_2_ ratio determined using a least-squares linear regression within the Ratiocalc Software (*54*) and multiplied by our SO_2_ flux, is 1850 ± 1960 tons day^−1^ (table S3). This is lower than the hot gas CO_2_ flux we calculate, albeit with notably larger errors and is 3 to 5x less than the overall CO_2_ flux presented here. This demonstrated that the typical approach of monitoring CO_2_ using MultiGAS and the methods for calculating flux will systematically undersample the CO_2_ emissions from low-temperature fumaroles where scrubbing reactions occur.

We have successfully deployed a new OMG analyzer in combination with a SO_2_ UV spectrometer to measure the gas plume composition and fluxes at SHV in Montserrat. The OMG consists of three separate analyzers, namely, a new UV spectrometer for SO_2_ and a mid-IR spectrometer for HCl, as well as a near-IR spectrometer for CO_2_, all containing a multipass cell for increased sensitivity. By traversing the plume at SHV using a helicopter, we reveal a spatially heterogeneous plume due to the shallow hydrothermal system of the volcano scrubbing the more soluble SO_2_ and HCl. The high frequency and response time of the OMG combined with the known location and temperatures of the individual fumaroles on the volcano allowed for structures within each plume to be ascribed to individual fumaroles. The HCl measured in the plume originates entirely from the four hottest fumaroles—Gas Vent, Headwall, Tar River Cliff, and Tar River Summit—which have temperatures between 300° and 500°C. Similarly, SO_2_ emissions are dominantly sourced from these fumaroles, with an extension of diffuse contributions to the plume from cooler fumaroles, where all HCl is scrubbed. In contrast, the CO_2_ emissions at Soufrière Hills are unaffected by hydrothermal scrubbing and are abundantly emitted by cooler summit fumaroles alongside the four hot fumaroles, as well as contributions of cold fumaroles on the flanks of the volcano, where all SO_2_ or HCl is lost to scrubbing reactions. We report a SO_2_ flux of 4.0 ± 0.9 kg s^−1^ using a UV spectrometer and CO_2_ and HCl fluxes for the hot fumarole emissions to be 28 ± 13 and 0.46 ± 0.11 kg s^−1^, respectively. HCl/SO_2_ ratios are in line with previous quiescent periods of lava extrusion and continue to be an important monitoring tool for changes in the dome activity. We use three different approaches to estimate the overall CO_2_ emission from Soufrière Hills at 61 ± 40 to 131 ± 66 kg s^−1^, two of which are geometrically based and make assumptions upon plume shape and a fixed cross-sectional concentration. We demonstrate that quantification of the cold CO_2_ emissions would not be possible using a standard MultiGAS instrument and that the widely used CO_2_/SO_2_ x SO_2_ flux quantification of CO_2_ flux would lead to over a threefold reduction in CO_2_ flux. However, to more accurately quantify the true CO_2_ output, we would require a full vertical raster of the plume using the OMG. Regardless, we demonstrate that Soufrière Hills is emitting substantially more CO_2_ than previously reported, which may carry over onto other hydrothermal capped volcanoes worldwide and so we may be systematically underestimating current volcanic CO_2_ budgets globally. The process of producing hot and cold CO_2_ emissions most likely disproportionally affects volcanoes with lower SO_2_ emissions. It is noted that, although we believe that, previously, we may have been systematically underestimating CO_2_ emission from hydrothermally capped volcanoes globally, we do not infer that this is a dominant driver of the current warming of the Earth’s atmosphere. Assuming that the overall CO_2_ flux represents the magmatic degassing at depth and using the hot CO_2_/(SO_2_/HCl), we determine the percentage of the SO_2_ and HCl lost to the hydrothermal system to be between 33–68 and 56–79%, respectively. The OMG instrument provides a fundamental advancement in multigas analyzers, offering an opportunity to improve our understanding of volcanic degassing from individual volcanoes and globally.

## MATERIALS AND METHODS

### Flight path

A vertical column sky-viewing UV spectrometer was used to measure the SO_2_ flux, and an OMG was used to measure in situ plume compositions during helicopter traverses below and within the plume at Soufrière Hills. Data were collected on 18 and 19 May 2017. The OMG experienced interference from helicopter vibrations on 18 May, and so the data presented here are from 19 May only. The UV spectrometer ran continuously throughout the duration of the flight, which began at 11:20 a.m. (local time), making a series of traverses at varying altitudes and distances from the volcano summit. To measure SO_2_ vertical column densities, the helicopter was flown ~200 feet (~60.96 m) above the deck (land or sea) to record as much of the volcanic plume as possible. Ten traverses beneath the plume were successfully conducted at distances of 2, 3.5, 4.5, and 6 km from the volcano summit using the UV spectrometer. To obtain in-plume gas compositions using the OMG, five traverses with the helicopter were recorded flying through the plume 2 km downwind of the summit. The average speed of the helicopter during the traverses was 30 m s^−1^.

### UV spectrometer

An Ocean Optics USB 2000+ spectrometer was connected to a vertically pointed collimating telescope (focal length = 10 cm), via a fiber optic cable. The spectrometer was controlled and powered by a USB connected to a laptop running the iFit package (*44*) (https://github.com/ManchesterVolcanology/iFit). The fit included absorption cross sections for SO_2_ (*58*) and O_3_ (*59*), as well as a Ring spectrum generated using the QDOAS software (*60*). The Fraunhofer reference spectrum used was from Chance and Kurucz (*61*). The window for fitting was between 310 and 320 nm, and stray light was removed between 280 and 290 nm. The instrument line shape was modeled as a super-Gaussian function, with the line shape parameters fitted as part of the retrieval. All spectra were corrected for dark current before analysis. Last, a wavelength shift and squeeze were fitted to allow for errors in the wavelength calibration of the spectrometer. The emission rate was calculated within the program by selecting appropriate windows for each traverse and using a wind speed of 10 m s^−1^ with an error of 20%. Wind speeds were measured in flight, flying at plume altitude by comparing the ground and air velocities of the helicopter.

### Optical MultiGas

The gas composition measurements described here were collected with an OMG consisting of three optical analyzers for in situ simultaneous measurements of five volcanic gases. The OMG includes IR spectrometers and one UV spectrometer in combination with multipass cells. Simultaneous measurements of CO_2_, HF, H_2_O, HCl, and SO_2_ absorption spectra are collected, with a time resolution better than 1 s. Each spectrum is then analyzed to produce concentrations using a simple fitting procedure, using measured local temperature and pressure and the HITRAN spectral line database (*62*) to define the absorption coefficients of each line. The OMG results for SO_2_ and HCl here have had no postfiltering or smoothing applied, and a Savitzky-Golay filter was applied to the CO_2_, with a fitting window of 7 and a second-order polynomial. The high sampling rate is essential due to the fast traverse speed to capture the details of compositional structure within the plume, which would otherwise be lost. The three sensors are attached mechanically to each other, but each spectrometer works independently and is equipped with a dedicated power supply and electronics, which control operations. Each system has its own internal clock and records the universal time.

During the measurements reported here, the OMG was installed on the aircraft and the three open-path multipass cells (path length of 20 m) were inserted into a plastic box, with external air funneled into and out of the box through a plastic pipe (5 cm in internal diameter), driven by dynamic pressure during the flight. This guaranteed the simultaneous analysis of the different gases while minimizing the possible loss of molecules sticking on the walls. A detailed description of this spectrometer is reported in (*63*). With respect to the sensor of Chiarugi *et al.* (*63*), the diode laser resonant to the CO_2_ absorption was replaced by a different source. A brief description of the optical spectrometers used in this study is reported in the Supplementary Materials.

### Gas composition calculations

To define the start and end of the plume for each traverse, we established a set of requirements: A minimum of 20 data points must be present, with no more than three consecutive values falling below the background concentration. In addition, the peak amplitude must exceed a value three times greater than the SD above the background concentration (Fig. 4). A final verification of the results is undertaken to ensure that plumes have not been identified as being present while the helicopter is turning around for the next traverse.

The CO_2_/(SO_2_/HCl) ratio for the central plume are derived by dot-to-dot comparison using the peak values in the SO_2_ and HCl traverses ascribed to the individual fumarole sources (17 and 20 values, respectively) (see Fig. 6). Uncertainties are propagated through the calculation using the SD of each gas as follows

$$∆\Phi =\Phi \sqrt{{\left(\frac{\sigma_{a}}{a}\right)}^{2}+{\left(\frac{\sigma_{b}}{b}\right)}^{2}}$$ (4)

where Φ is the molar ratio of gases a and b, σ is the SD, and ∆Φ is the molar ratio error. We calculate the HCl/SO_2_ molar ratio for the central plume by fitting an orthogonal distance regression line to scatterplots of HCl and SO_2_ (fig. S3). We include the SO_2_ edge values in this regression as they are required to calculate a true HCl flux due to multiplying by the SO_2_ flux obtained from the UV spectrometer. The error for the fitment of the regression line is based on a 95% confidence interval and includes the SD of each measurement in its calculation.

To calculate the overall gas composition from Soufrière Hills, we must include the wider CO_2_ emissions where SO_2_ and HCl are at background levels. We provide three different approaches to estimate the composition and flux. The first method determines the gas mixing ratios by integrating the sum of each gas species above background concentrations over the plume width of CO_2_. The absolute error (Δ) for the integrated area (A) of a single gas species (a) for each traverse is calculated by the following

$$\Delta A_{a}=n\;\sqrt{{\left(\sigma_{a}\right)}^{2}}$$ (5)

where n is the number of data points and σ is the SD of the gas species a. To obtain the error of the molar ratios (Φ) between the integrated areas of two gas species, a and b, we use the following

$$\Delta \Phi =\Phi \sqrt{{\left(\frac{\Delta A_{a}}{A_{a}}\right)}^{2}+{\left(\frac{\Delta A_{b}}{A_{b}}\right)}^{2}}$$ (6)

After achieving a molar ratio and absolute error from each of the methods, we must calculate the flux (J) for each gas. To calculate the flux for CO_2_ and HCl, we must convert the molar ratio and error of (CO_2_/HCl) /SO_2_ to a mass ratio (ε) and then multiply this value by the SO_2_ flux derived from the UV spectrometer. We propagate the errors in this calculation using the following

ΔJa=Ja(Δεε)2+(ΔJSO2JSO2)2 (7)

The second method uses a geometric approach to determine the gas mixing ratio for the overall emissions at Soufrière Hills. This method requires us to use the average SO_2_ concentration and known width of our plume to calculate the cross-sectional area required to match the SO_2_ flux as measured by the UV spectrometer. We achieve this by using the ideal gas law to convert the average concentration along our traverse from ppm to mass per unit area. We can then use this value, with the known width of the plume to calculate the height of the plume that produces the cross-sectional area required to equal the SO_2_ flux obtained from the UV spectrometer. We calculate the mass per unit area of CO_2_ and HCl as we did for SO_2_. Next, the average plume width for CO_2_ and HCl are multiplied by the newly calculated height of the SO_2_ plume to calculate their cross-sectional area, which, in turn, is multiplied by their average concentration to produce a flux. This series of calculations assumes that the vertical concentration of gas in the plume is fixed. The calculation requires the height of the different gas plumes to be the same, assumed to be thermally constrained but allows for lateral spreading above our traverse intercept by varying the height to retain the cross-sectional area required to produce the desired flux (fig. S4A). The error for each flux is calculated using the ideal gas law and the molar mass of the gas species to convert the SD of the gas concentration into a mass error per unit area. This value is then multiplied by the calculated area of each plume to determine the flux error. After calculating the different flux for each gas, we can calculate the mixing ratios for this method and its error.

To test the sensitivity of the optimized geometry method to differences in thermal buoyancy between the broader, cooler flank emissions of CO_2_ and the hotter, central CO_2_ emissions, we recalculated the approach by dividing the CO_2_ into two regions, each with its own concentration. In this recalculation, we assume that the CO_2_ plume height decreases beyond the width of SO_2_, which we use as a proxy for the central hot fumarole width. The results of this analysis are shown graphically in fig. S4B. The CO_2_ flux calculated using this second approach falls within the error of the original geometric approach, demonstrating that the CO_2_ flux is not notably affected by variations in the shape of the broader CO_2_ emissions.

The third method uses the same geometric-based approach as method two but instead assumes that our traverse flew through the center of a circular plume, and so our measured width for each gas plume is equal to the diameter of that plume. This method adopts the same assumptions as method two. However, as the traverse was conducted at ~350 m above sea level, this would result in a substantial portion of the plume being placed underground (fig. S4C). The error for this method is calculated using the same approach as was described for the optimized geometric approach.

## Supplementary Materials

### This PDF file includes:

Supplementary Text Figs. S1 to S10 Tables S1 to S6 References
