# Supplementary material for: Quantification of low-temperature gas emissions reveals CO2 flux underestimates at Soufrière Hills volcano, Montserrat
Source: Sci Adv. 2025 Apr 2;11(14):eads8864. doi: 10.1126/sciadv.ads8864 (PMC11963986; doi:10.1126/sciadv.ads8864)
Supplement: Supplementary file 1 — Supplementary Text Figs. S1 to S10 Tables S1 to S6 References [file sciadv.ads8864_sm.pdf]

Science Advances
AAAS

Supplementary Materials for

Quantification of low-temperature gas emissions reveals CO_2 flux underestimates at Soufrière Hills volcano, Montserrat

Alexander Riddell *et al.*

Corresponding author: Alexander Riddell, alexander.riddell@manchester.ac.uk

Sci. Adv. **11**, eads8864 (2025)
DOI: 10.1126/sciadv.ads8864

This PDF file includes:

Supplementary Text
Figs. S1 to S10
Tables S1 to S6
References

Supplementary Text

Optical Multigas description - IR spectrometers

The IR spectrometers are based on the "Direct Absorption" (DA) technique: the emission wavelengths of two diode lasers are scanned respectively across the selected absorption lines of the target molecules, by modulating the laser current with a ramp signal. The concentration is calculated by fitting the detected absorption signal, recorded at the exit of the cell, knowing the multipass path length, the molecule line strength (according to the HITRAN2020 molecular database (62)) and pressure and temperature of the sampled air.

A scheme of the setup for both analysers is shown in **fig. S5**. The area within the dashed line is sealed against the corrosive atmosphere which can be found aside from volcanos. Two laser beams are combined onto a beam splitter. One of the combined beams is sent, along a unique optical path, to a custom-built Herriott multipass cell, with a total path length of 20.23 m in 52 passes. This occurs on one side of the optical bench. The other combined beam (reference signals) and the combined beam exiting from the cell (measurement signals) are sent to the other side of the optical bench and recorded by two detectors. An etalon and a reference cell, containing the target molecules, are placed along the path of the reference beams to obtain a frequency reference for the linearisation and calibration of the frequency scale of the absorption signal and to check that the laser scan is set to the selected line absorption. The two lasers work in sequence and are switched on alternately, each one at the repetition frequency of 500 Hz. At the beginning of each ramp, there is a short (100 μs) period of no emission, to measure the electric zero of the detectors + amplifiers. The computer for control and acquisition is a CompactRIO crate by National Instruments (cRIO 9074), which combines a dual-core processor, a reconfigurable FPGA (field programmable gate array), and five commercial plugins for fast acquisition (four independent channels, 1MHz at 16 bits); slow acquisition (eight multiplexed channels, 500 kHz at 12 bits); digital I/O; thermocouple reading and data storage. The acquired spectra are averaged according to the selected integration time and saved.

The CO_2 and HF analyser is based on fibre components and the beam splitter is a 2x2 fibre coupler. The two sources are near-IR distributed feedback (DFB) fibre-coupled diode lasers, emitting respectively at 2.004 μm (for CO_2 measurement) and at 1.278 μm (for HF measurement).

A detailed description of this spectrometer is reported in (64). With respect to the sensor of Chiarugi et al. (64), the diode laser resonant to the CO_2 absorption was replaced by a different source (NANOPLUS DFB-2004-4-BF2-FC/APC) with a higher tunability (≥ 1.5 cm^{-1}) for the simultaneous measurement of CO_2 and H_2O. The laser can operate in two different settings: (i) for the detection of CO_2 only, by scanning around 4991.26 cm^{-1} (2003.50 nm) and (ii) for the detection of CO_2 and H_2O, by scanning around the absorptions of CO_2 at 4992.52 cm^{-1} (2003.00 nm) and H_2O at 4992.94 cm^{-1} (2002.83 nm). The diode laser for HF measurement is the same described in Chiarugi et al. (63) and the laser scan is around the HF absorption at 7823.82 cm^{-1} (1278.15 nm).

The CO_2 in-flight stability was estimated as twice the standard deviation of the concentration when the aircraft is outside the volcanic plume, where we can assume that CO_2 is constant. The CO_2 long-term stability results of 2 ppm when the scan includes only CO_2 and it is

reduced to 4 ppm during the simultaneous measurement of CO_2 and H_2O, due to the overlapping of the two absorption lines which decreases the performance of the post-processing analysis. For this reason, during the measurement described in the present paper, the laser was set to scan only the CO_2 absorption. The characteristics and performance of the CO_2, HF, H_2O spectrometer is reported in **table S.4**.

The HCl analyser is a free-space mid-infrared spectrometer developed for the simultaneous measurement of the isotopologues HCl^{35} and HCl^{37}, to examine feasibility of HCl isotopic ratio measurements. The sensor is based on two Mid InfraRed diode lasers (NANOPLUS GmbH DFB, TO5 with TEC and window), emitting around 3.4 µm and scanning on the HCl^{37} absorption at 2942.72 cm^{-1} (3398.21 nm) and on the HCl^{35} absorption at 2944.91 cm^{-1} (3395.68 nm), respectively. The solution with two sources was chosen because the absorptions of the two isotopologues are about 2.2 cm^{-1} away from each other, so it's challenging to encompass both in a unique laser scan. A picture of the HCl analyser is shown in **fig.S6**, where the laser side is on the right, and the detector side is on the left.

Typical HCl spectra, recorded with the HCl analyser in open-path configuration during a ground-based in-situ test campaign on Etna volcano, Italy, corresponding to a concentration of 2630 ppb for HCl^{35} and of 852 ppb for HCl^{37}, are shown in **fig. S7**.

To verify the reliability of the measured HCl concentration value, the spectrometer was tested in the laboratory by using an HCl mixture in the closed-cell configuration. The multipass cell was closed with a Teflon cylinder, and Teflon pipes and a pump were used to introduce the HCl mixture inside the cylinder. The mixture was prepared starting from pure HCl, and gradually diluting it with ambient air, down to a final concentration of 15.0 ± 1.5 ppm. The dilution was calculated based on the pressures read by the pressure transducers (10 Torr F.S. and 1000 Torr F.S.). The 10 Torr gauge was used at the beginning for HCl and the 1000 Torr gauge for all the other readings. The test was performed in a thermal chamber at around 25 ^{0}C. HCl concentration was retrieved according to the procedure described by Chiarugi et al. (63). The concentration uncertainty is determined by combining the accuracy of the temperature and pressure measurements (respectively 1 and 0.8 %), the accuracy of the HCl^{35} line strength according to the HITRAN2020 database (2 %) (*63*, 64) and the uncertainty in the fitting procedure (0.2 %). The resulting total accuracy is 4 %. The mean value of the HCl concentration, obtained after 2 hours of measurement at an integration time of 1.5 s, is 15.5 ppm, which, considering an accuracy of 0.6 ppm (4%), agrees with the mixture concentration.

To evaluate the ultimate sensitivity of the HCl spectrometer, an Allan–Werle variance analysis of the obtained concentration was carried out. An Allan–Werle variance plot of the HCl concentration is presented in **fig. S8** as a function of the integration time. A sensitivity of about 3 ppb is obtained, for an integration time of 1.5 s. The best achievable sensitivity, better than 2 ppb, can be reached for 5 s of integration time. The characteristics and performance of the HCl spectrometer are reported in **table S.5**.

Optical Multigas description - UV spectrometer

The SO_2 analyser is based on direct absorption in UV wavelengths. A fibre-coupled LED, emitting in the range 300-315 nm (with a Full Width Half Maximum of 12 nm), mounted on one side of the optical bench, is sent to a custom-made Herriott multipass cell, with a total path

length of 20.23 m in 52 passes. The cell is equal to the two of the IR spectrometers, the only difference being in the mirror optical coating, tailored for UV. The signal exiting from the multipass cell is sent to the other side of the optical bench and is detected by a spectrometer with a resolution of 0.4 nm (Ocean Optics USB2000+; 2048-element CCD-array). A Raspberry Pi system is used for control and acquisition. A picture of the SO_2 analyser is shown in **fig. S9**, where the led side is on the left, and the spectrometer side is on the right. A typical 10 ppm-SO_2 absorption spectrum recorded by the sensor is shown in **fig. S10**.

The SO_2 concentration is obtained as the least squares solution that minimises the difference between the measured spectra (divided by the multipass path length) and the tabulated SO_2 cross-section according to (65). The concentration accuracy is determined by combining the uncertainty of the known cross section (3 %) and the uncertainty of the least-squares algorithm (1%). The characteristics and performances of the SO_2 spectrometer are reported in **table S.6**.

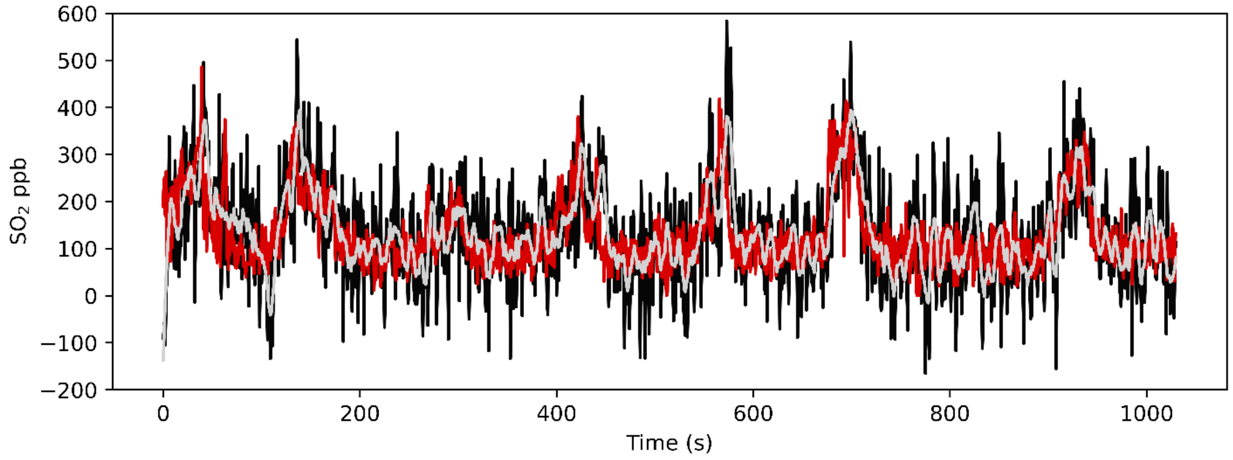

fig. S1.

fig. S1. Resampling of the OMG SO_2 signal using the characteristics of the multiGAS sensor. The original OMG SO_2 signal is shown by the red line, the black line represents the original OMG SO_2 signal using the SO_2 sensor characteristics of Liu et al. (53) without smoothing and the grey line is the black line processed in Ratiocalc, where a Savitzky-Golay filter, window length 13, 2nd order polynomial smoothing function was applied. This shows that the narrow SO_2 peaks produced from individual hot fumaroles observed with OMG are likely to have been not revealed by a standard MultiGas SO_2 instrument.

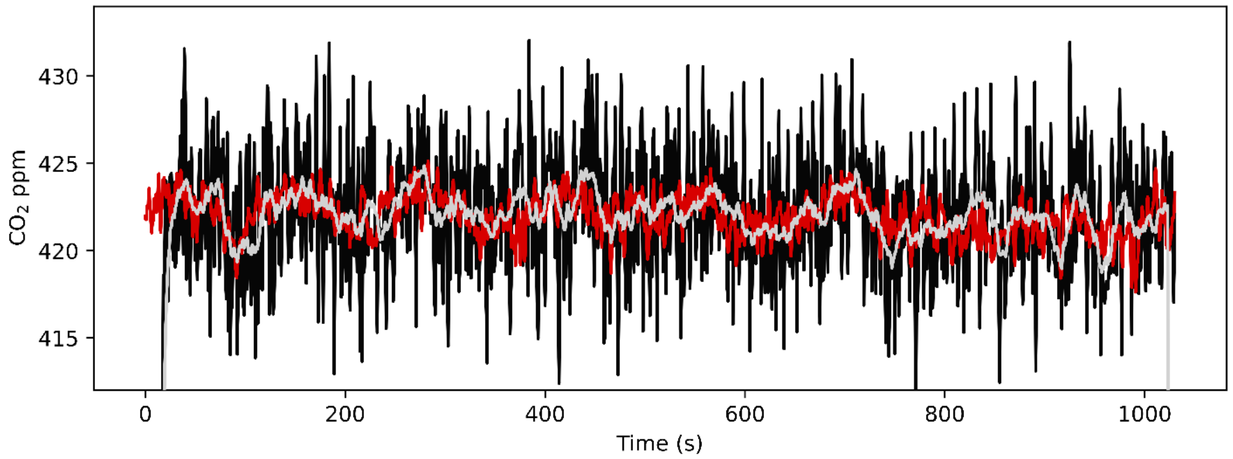

fig. S2.

fig. S2. Resampling of the OMG CO_2 signal using the characteristics of the multiGAS sensor. The original OMG CO_2 signal is shown by the red line, the black line represents the original OMG CO_2 signal using the CO_2 sensor characteristics of Liu et al. (53) without smoothing and the grey line is the black line with a 15 second N-point moving average applied. This shows that the modest CO_2 enhancement observed with OMG are likely to have been not revealed by a standard MultiGas CO_2 instrument.

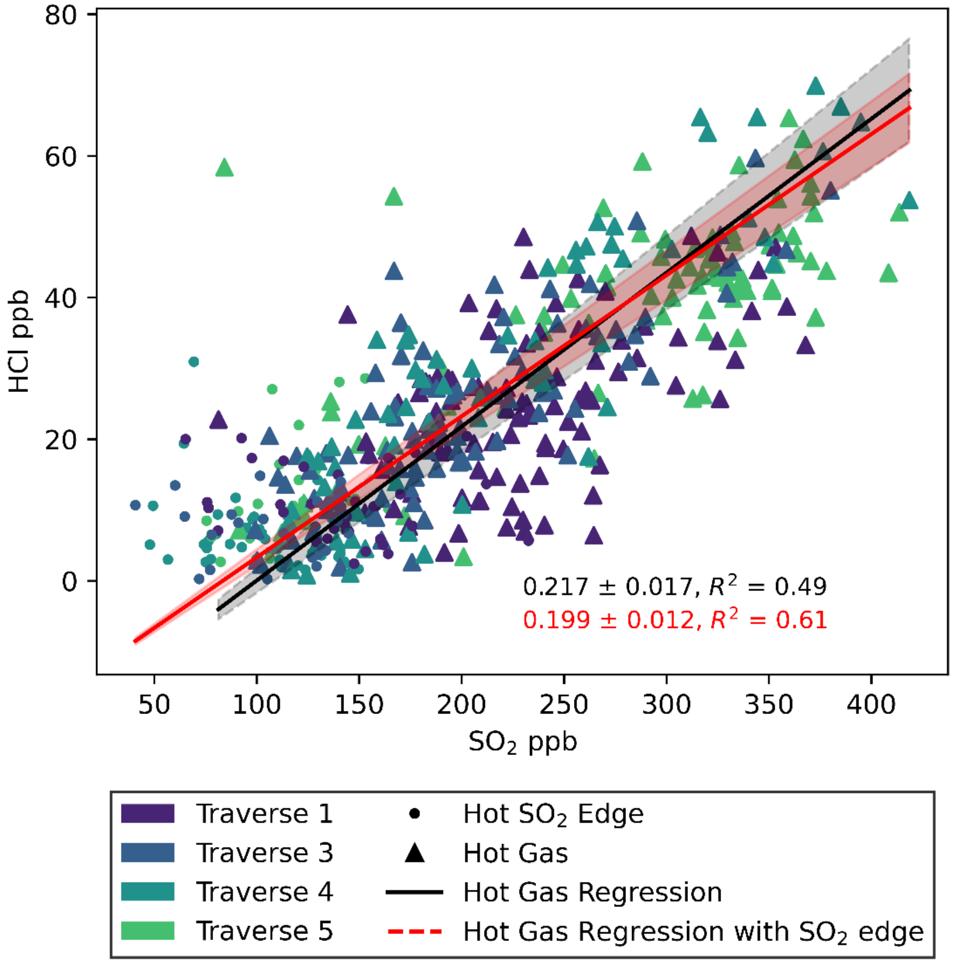

fig. S3.

fig. S3. HCl/SO_2 hot gas correlation plots. An orthogonal distance regression (ODR) line is calculated for the HCl/SO_2 values over the plume width of HCl (black line), and a second ODR line is plotted that includes the 'SO_2 edge' values and the corresponding background HCl (red line). The correlation coefficient and regression coefficient are displayed for the two different regression lines. The error for the regression coefficient is calculated with a 95% confidence interval, the standard deviation of each measurement is accounted for in the ODR fitment.

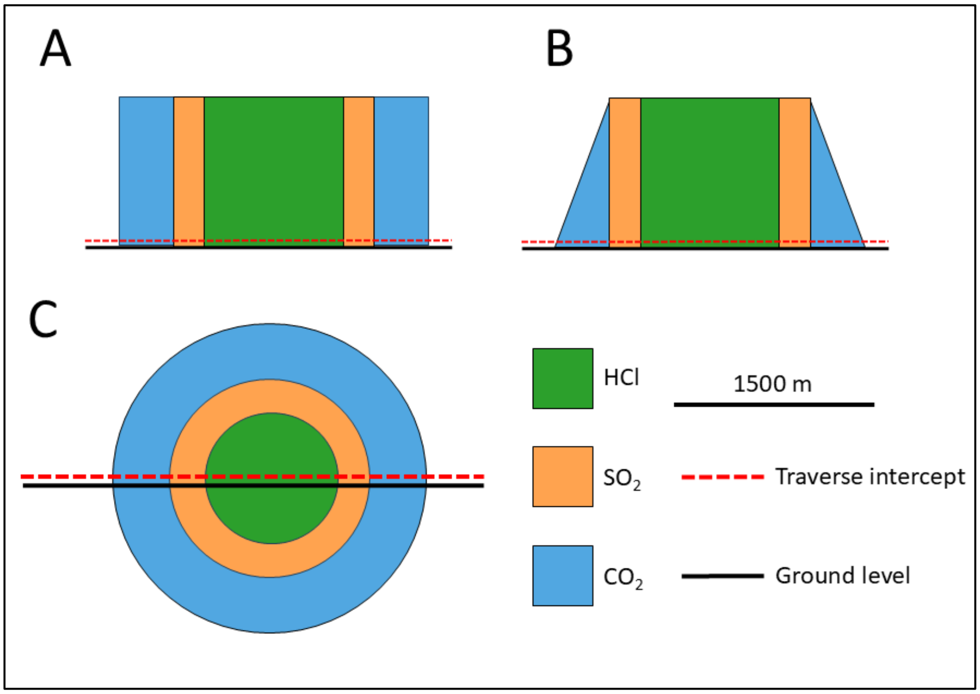

fig. S4.

fig. S4. Geometric approaches for estimating overall plume flux. (A) Method two for estimating the cold CO_2 flux using the optimised geometric approach and maintaining a constant CO_2 concentration across the entire width of the plume and all gases sharing the same plume height. **(B)** Method two for estimating the cold CO_2 flux using the optimized geometric approach but dividing the CO_2 into two regions: a central sector (with the width of the SO_2 plume) where CO_2 concentrations are higher, and an outer region with a lower CO_2 concentration and where the plume height decreases due to cooler sources with lower thermal buoyancy. This method yields the same SO_2 and HCl fluxes as in Panel (A), but results in a CO_2 flux of 4975 ± 2460, which is within the error of the CO_2 flux calculated in Panel (A). **(C)** Method three is used to estimate the cold CO_2 flux using the circular plume geometry. The red dashed line represents the intercept of the plume by the helicopter, while the black line indicates the average distance to the ground below on all figures. All figures are drawn to scale.

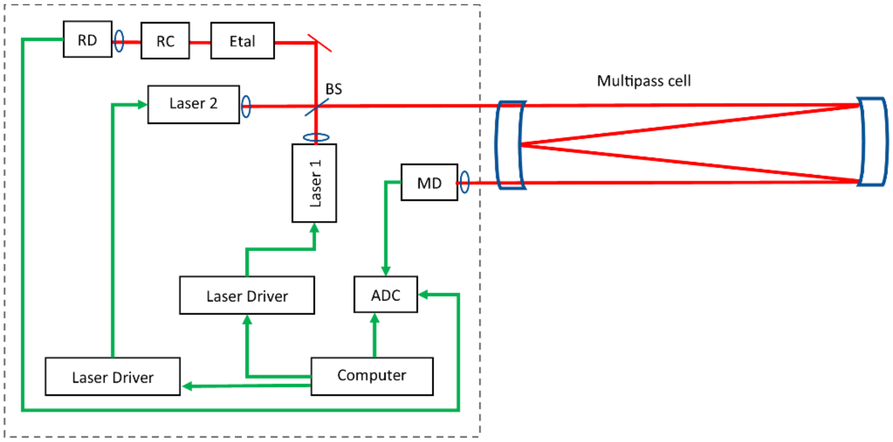

fig. S5.

fig. S5. Sketch of the apparatus for both IR-spectrometers: RD Reference Detector, RC Reference Cell, Etal Etalon, BS Beam Splitter, MD Measurement Detector. The dashed line encompasses the safe area.

fig. S6.

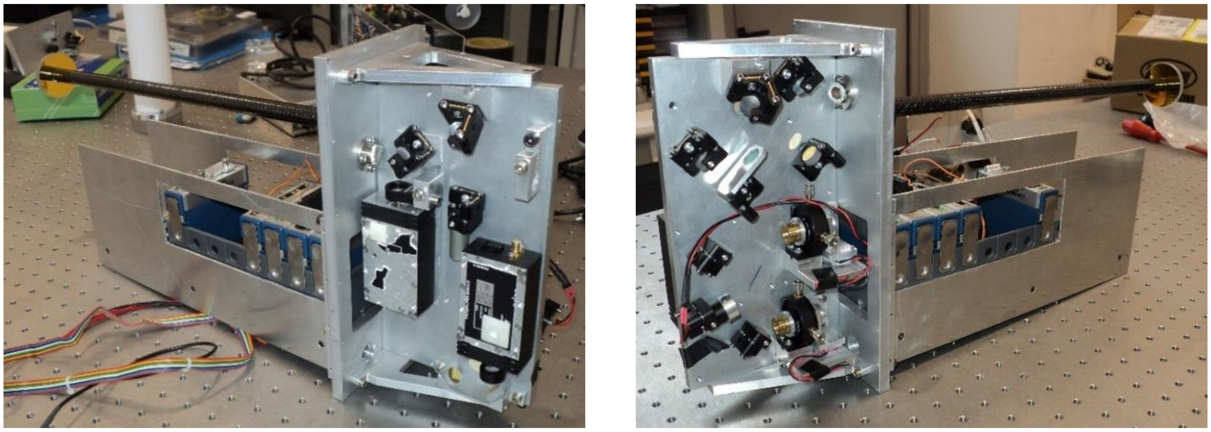

fig.S6. Photo of the HCl analyser. Lasers side is on the right, and the detectors side is on the left.

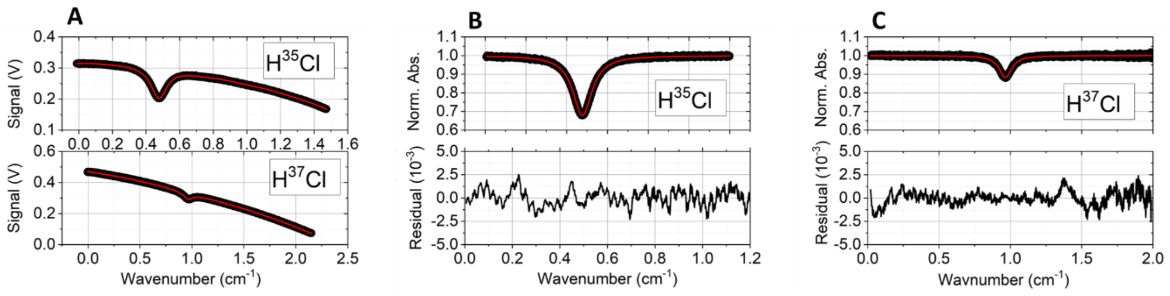

fig. S7.

fig. S7. Fitted spectra for the two HCl isotopologues. (A) Typical HCl spectra, corresponding to a concentration of 2630 ppb for HCl^{35} and of 852 ppb for HCl^{37}, were recorded during a test campaign on Etna volcano (NE crater) at a pressure of 0.7 atm and a temperature around 10 ^{0}C. **(B)** and **(C)** Normalised absorption spectra with fitting procedure results and corresponding residual (Black circle = experimental data. Red line = line fit according to Voigt function).

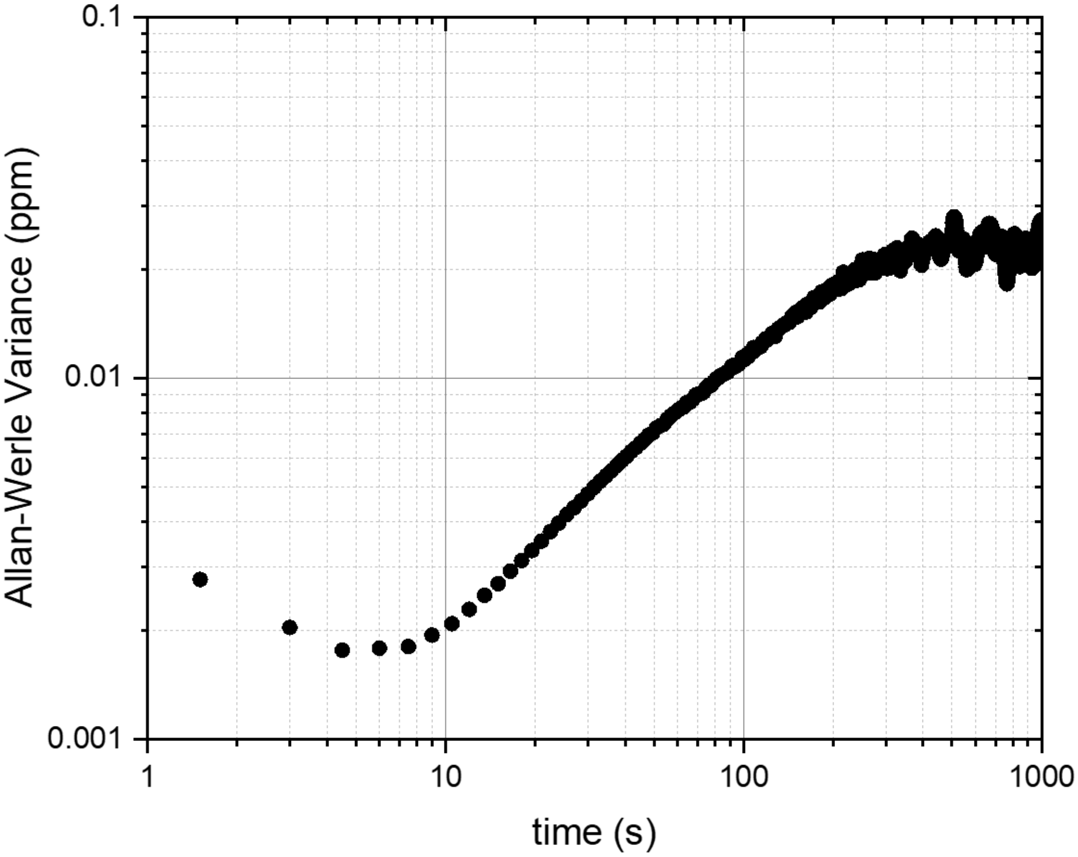

fig. S8.

fig. S8. Allan–Werle variance plot of 2 h in-flow measurements of an HCl calibrated mixture. The mixture is about 15 ppm in air, at an integration time of 1.5.

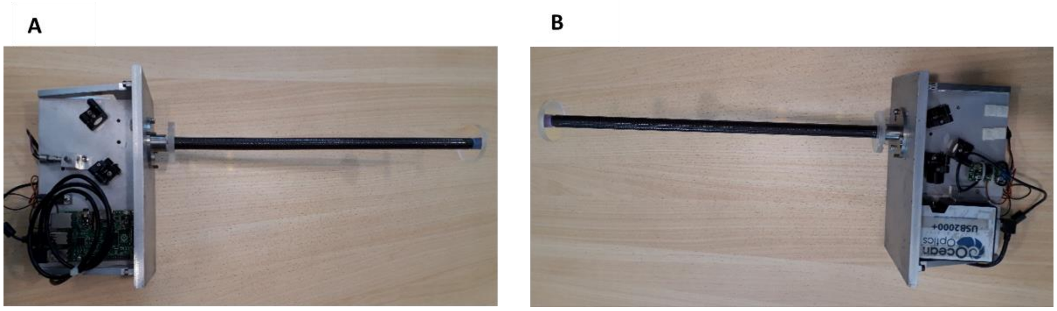

fig. S9.

fig. S9. Photo of the SO_2 analyser: LED side on the left, spectrometer side on the right.

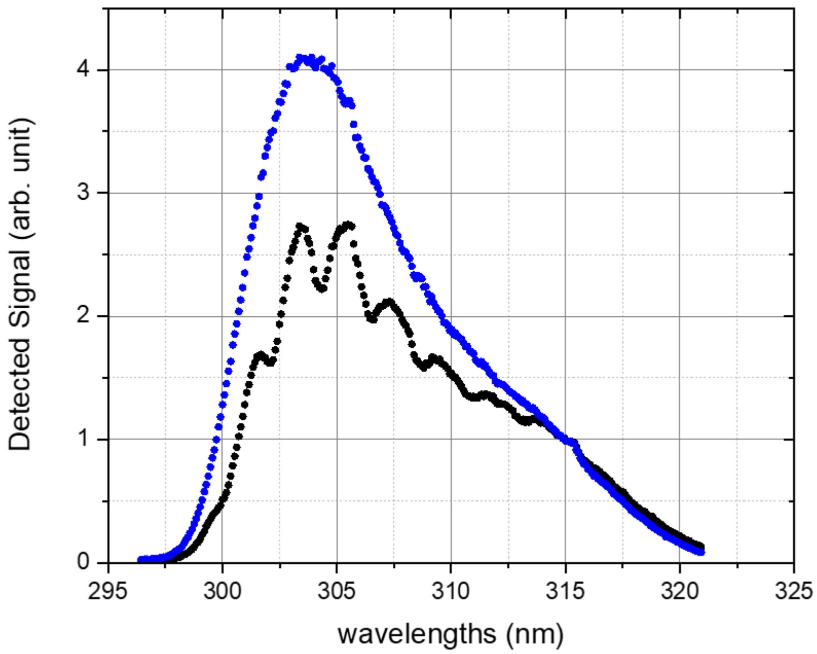

fig. S10.

fig. S10. UV absorption spectrum. UV spectrum of the LED source without absorption (blue line) and with 10ppm-SO_2 absorption (black line).

table S1.

table S.1. Calculated SO_2 emission. SO_2 emission rate calculated using UV spectrometer data analysed using iFit and a wind speed of 10 $m \cdot s^{-1}$. Error is a combination of random error due to the fitting of spectra within iFit and more importantly due to the uncertainty of the wind measurements.

Distance from Summit of Volcano	SO_2 Flux ($kg \cdot s^{-1}$)	Number of traverses
2 km	4.0 ± 0.7	4
3.5 km (Top of Plymouth)	4.1 ± 1.0	2
4.5 km (Shoreline)	4.5 ± 1.1	2
6 km (At Sea)	3.6 ± 1.1	2
Weighted Average	4.0 ± 0.9	10

table S2.

table S.2. Optical MultiGAS vs MultiGAS comparison. A comparison of the sensor measurement time and accuracy of those described in this paper and other gas sensors used in MultiGAS publications.

Ref.	CO_2	Time Res. (s)	Accuracy	SO_2	Time Res. (s)	Accuracy	HCl	Time Res. (s)	Accuracy	HF	Time Res. (s)	Accuracy	H_2O	Time Res. (s)	Accuracy
This paper	Laser Spectr.	0.5	4% read.	LED Spectr.	0.5	4% read.	Laser Spectr.	0.5	4% read.	Laser Spectr.	0.5	4.5% read.	Laser Spectr.	0.5	4% read.
(19)	NDIR Spectr.	9	1.5% read.	Electrochem. sensor	9	2% read.							NDIR Spectr.	9	1.5% read.
(20)	NDIR Spectr.	3	2% range	Electrochem. sensor	20	2% read.									
(21)	NDIR Spectr.	15	1.5% read.	Electrochem. sensor	30	20% read.							Capacitive	5	2% RH

table S3.

table S.3. CO_2/SO_2 molar ratio exported from Ratiocalc using the MultiGAS simulated data sets. These include a 15s moving average applied to the CO_2 dataset and using the Savitzky-Golay smoothing function within Ratiocalc for SO_2 (Window length – 13, polynomial – 2). CO_2 flux is calculated using the converted mass ratio and using the SO_2 flux from the UV spectrometer of 348 ± 78 tonnes per day.

Traverse	CO_2/SO_2 molar ratio	CO_2 Flux ($t \cdot day^{-1}$)
1	8 ± 7	1910 ± 1730
3	8 ± 11	1910 ± 2660
4	6 ± 8	1430 ± 1940
5	9 ± 6	2151 ± 1513
Average	8 ± 8	1850 ± 1960

table S4.

table S.4. Characteristics and performance of the CO_2+HF+H_2O spectrometer.

Near-IR spectrometer for CO_2+HF+H_2O	
Dimension	600x132x276 mm^3
Weight	8 Kg
Power consumption	30 W @14V
Acquisition Rate	4 Hz (maximum) - 2 Hz (Montserrat measurement)
Sensitivity (@ integration time = 0.5 s)	0.4 ppm for CO_2<br>25 ppb for HF<br>0.1 % for H_2O
Accuracy	4% for CO_2<br>4.5 % for HF<br>4% for H_2O

table S5.

table S.5. Characteristics and performance of the HCl spectrometer.

Mid-infrared spectrometer for HCl	
Dimension	589x122x266 mm^3
Weight	9 Kg
Power consumption	40 W @12V
Acquisition rate	4 Hz (maximum) - 2.5 Hz (Montserrat measurement)
Sensitivity (@ integration time = 0.4 s)	10 ppb
Accuracy	4%

table S6.

table S.6. Characteristics and performance of the SO_2 spectrometer.

UV spectrometer for SO_2	
Dimension	620x132x226 mm^3
Weight	2.5 Kg
Power consumption	10 W @5V
Acquisition rate	4 Hz (maximum) - 2 Hz (Montserrat measurement)
Sensitivity (@ integration time = 0.5 s)	200 ppb
Accuracy	4%

REFERENCES AND NOTES

1. S. K. Brown, S. C. Loughlin, R. S. J. Sparks, C. Vye-Brown, J. Barclay, E. Calder, E. Cottrell,G. Jolly, J.-C. Komorowski, C. Mandeville, C. G. Newhall, J. L. Palma, S. Potter, G. Valentine, "Global volcanic hazard and risk" in *Global Volcanic Hazards and Risk*, C. Vye-Brown, S. K. Brown, S. Sparks, S. C. Loughlin, S. F. Jenkins, Eds. (Cambridge Univ. Press, 2015), pp. 81–172.

2. A. Hooper, F. Prata, F. Sigmundsson, Remote sensing of volcanic hazards and their precursors. *Proc. IEEE* **100**, 2908–2930 (2012).

3. A. Aiuppa, R. Moretti, C. Federico, G. Giudice, S. Gurrieri, M. Liuzzo, P. Papale, H. Shinohara, M. Valenza, Forecasting Etna eruptions by real-time observation of volcanic gas composition. *Geology* **35**, 1115–1118 (2007).

4. S. Valade, A. Ley, F. Massimetti, O. D'Hondt, M. Laiolo, D. Coppola, D. Loibl, O. Hellwich, T. R. Walter, Towards global volcano monitoring using multisensor sentinel missions and artificial intelligence: The MOUNTS monitoring system. *Remote Sens.* **11**, 1528 (2019).

5. S. A. Carn, "Gas, plume, and thermal monitoring" in *The Encyclopedia of Volcanoes* (Elsevier, 2015), pp. 1125–1149.

6. J. M. de Moor, A. Aiuppa, J. Pacheco, G. Avard, C. Kern, M. Liuzzo, M. Martínez, G. Giudice, T. P. Fischer, Short-period volcanic gas precursors to phreatic eruptions: Insights from Poás Volcano, Costa Rica. *Earth Planet. Sci. Lett.* **442**, 218–227 (2016).

7. J. M. de Moor, A. Aiuppa, G. Avard, H. Wehrmann, N. Dunbar, C. Muller, G. Tamburello, G. Giudice, M. Liuzzo, R. Moretti, V. Conde, B. Galle, Turmoil at Turrialba Volcano (Costa Rica): Degassing and eruptive processes inferred from high-frequency gas monitoring. *J. Geophys. Res. Solid Earth.* **121**, 5761–5775 (2016).

8. A. S. Daag, M. T. Dolan, E. Laguerta, G. P. Meeker, C. Newhall, J. Pallister, R. U. Solidum, "Monitoring sulphur dioxide emissions at Mount Pinatubo, Philippines" in *Fire and Mud: Eruptions and Lahars of Mount Pinatubo* (Philippine Institute of Volcanology and Seismology, 1996), pp. 647–664.

9. T. Christopher, M. Edmonds, B. Taisne, H. Odbert, A. Costa, V. Hards, G. Wadge, Periodic sulphur dioxide degassing from the Soufrière Hills volcano related to deep magma supply. *Geol. Soc. Spec. Pub.* **410**, 123–141 (2015).

10. S. Giammanco, G. Pecoraino, Geochemical characterization and temporal changes in parietal gas emissions at Mt. Etna (Italy) during the period July 2000–July 2003. *Terr. Atmos. Ocean. Sci.* **16**, 805 (2005).

11. M. L. Carapezza, S. Inguaggiato, L. Brusca, M. Longo, Geochemical precursors of the activity of an open-conduit volcano: The Stromboli 2002–2003 eruptive events. *Geophys. Res. Lett.* **31**, L07620 (2004).

12. M. R. Burton, T. Caltabiano, F. Murè, G. Salerno, D. Randazzo, SO_2 flux from Stromboli during the 2007 eruption: Results from the FLAME network and traverse measurements. *J. Volcanol. Geotherm. Res.* **182**, 214–220 (2009).

13. C. Kern, A. H. Lerner, T. Elias, P. A. Nadeau, L. Holland, P. J. Kelly, C. A. Werner, L. E. Clor, M. Cappos, Quantifying gas emissions associated with the 2018 rift eruption of Kīlauea Volcano using ground-based DOAS measurements. *Bull. Volcanol.* **82**, 55 (2020).

14. T. Kagoshima, Y. Sano, N. Takahata, T. Maruoka, T. P. Fischer, K. Hattori, Sulphur geodynamic cycle. *Sci. Rep.* **5**, 8330 (2015).

15. T. Plank, C. E. Manning, Subducting carbon. *Nature* **574**, 343–352 (2019).

16. T. A. Mather, Volcanoes and the environment: Lessons for understanding Earth's past and future from studies of present-day volcanic emissions. *J. Volcanol. Geotherm. Res.* **304**, 160–179 (2015).

17. G. Stenchikov, "Chapter 29—The role of volcanic activity in climate and global changes" in *Climate Change (Third Edition)*, T. M. Letcher, Ed. (Elsevier, 2021), pp. 607–643.

18. S. A. Carn, L. Clarisse, A. J. Prata, Multi-decadal satellite measurements of global volcanic degassing. *J. Volcanol. Geotherm. Res.* **311**, 99–134 (2016).

19. A. Aiuppa, G. Giudice, S. Gurrieri, M. Liuzzo, M. Burton, T. Caltabiano, A. J. S. McGonigle, G. Salerno, H. Shinohara, M. Valenza, Total volatile flux from Mount Etna. *Geophys. Res. Lett.* **35**, L24302 (2008).

20. A. Aiuppa, C. Federico, G. Giudice, S. Gurrieri, Chemical mapping of a fumarolic field: La Fossa Crater, Vulcano Island (Aeolian Islands, Italy). *Geophys. Res. Lett.* **32**, L13309 (2005).

21. H. Shinohara, A new technique to estimate volcanic gas composition: Plume measurements with a portable multi-sensor system. *J. Volcanol. Geotherm. Res.* **143**, 319–333 (2005).

22. M. Edmonds, E. J. Liu, K. V. Cashman, Open-vent volcanoes fuelled by depth-integrated magma degassing. *Bull. Volcanol.* **84**, 28 (2022).

23. B. T. McCormick Kilbride, E. J. Nicholson, K. T. Wood, T. C. Wilkes, C. I. Schipper, K. Mulina, I. Itikarai, T. Richardson, C. Werner, C. S. L. Hayer, B. Esse, M. Burton, T. D. Pering, A. J. S. McGonigle, D. Coppola, M. Bitetto, G. Giudice, A. Aiuppa, Temporal variability in gas emissions at Bagana volcano revealed by aerial, ground, and satellite observations. *Geochem. Geophys. Geosyst.* **24**, e2022GC010786 (2023).

24. G. Tamburello, S. Moune, P. Allard, S. Venugopal, V. Robert, M. Rosas-Carbajal, S. Deroussi, G.-T. Kitou, T. Didier, J.-C. Komorowski, F. Beauducel, J.-B. De Chabalier, A. Le Marchand, A. Le Friant, M. Bonifacie, C. Dessert, R. Moretti, Spatio-temporal relationships between fumarolic activity, hydrothermal fluid circulation and geophysical signals at an arc volcano in degassing unrest: La Soufrière of Guadeloupe (French West Indies). *Geosciences* **9**, 480 (2019).

25. C. Werner, T. Hurst, B. Scott, S. Sherburn, B. W. Christenson, K. Britten, J. Cole-Baker, B. Mullan, Variability of passive gas emissions, seismicity, and deformation during crater lake growth at White Island Volcano, New Zealand, 2002–2006. *J. Geophys. Res. Solid Earth.* **113**, B01204 (2008).

26. R. B. Symonds, T. M. Gerlach, M. H. Reed, Magmatic gas scrubbing: Implications for volcano monitoring. *J. Volcanol. Geotherm. Res.* **108**, 303–341 (2001).

27. C. Werner, W. C. Evans, P. J. Kelly, R. McGimsey, M. Pfeffer, M. Doukas, C. Neal, Deep magmatic degassing versus scrubbing: Elevated CO_2 emissions and C/S in the lead-up to the 2009 eruption of Redoubt Volcano, Alaska. *Geochem. Geophys. Geosyst.* **13**, 2011GC003794 (2012).

28. M. Edmonds, C. Oppenheimer, D. M. Pyle, R. A. Herd, G. Thompson, SO_2 emissions from Soufrière Hills Volcano and their relationship to conduit permeability, hydrothermal interaction and degassing regime. *J. Volcanol. Geotherm. Res.* **124**, 23–43 (2003).

29. E. M. Syracuse, G. A. Abers, Global compilation of variations in slab depth beneath arc volcanoes and implications. *Geochem. Geophys. Geosyst.* **7**, 2005GC001045 (2006).

30. G. Wadge, B. Voight, R. S. J. Sparks, P. D. Cole, S. C. Loughlin, R. E. A. Robertson, "Chapter 1—An overview of the eruption of Soufrière Hills Volcano, Montserrat from 2000 to 2010" in *Memoirs* (The Geological Society, 2014), vol. 30, pp. 1–40.

31. R. S. J. Sparks, S. R. Young, The eruption of Soufrière Hills Volcano, Montserrat (1995-1999): overview of scientific results. *Geological Society, London, Memoirs 21*, 45–69 (2002).

32. M. D. Murphy, R. S. J. Sparks, J. Barclay, M. R. Carroll, T. S. Brewer, Remobilization of andesite magma by intrusion of mafic magma at the Soufriere Hills Volcano, Montserrat, West Indies. *J. Petrol.* **41**, 21–42 (2000).

33. J. Barclay, M. J. Rutherford, M. R. Carroll, M. D. Murphy, J. D. Devine, J. Gardner, R. S. J. Sparks, Experimental phase equilibria constraints on pre-eruptive storage conditions of the Soufriere Hills magma. *Geophys. Res. Lett.* **25**, 3437–3440 (1998).

34. J. W. Neuberg, B. Taisne, M. Burton, G. A. Ryan, E. Calder, N. Fournier, A. S. D. Collinson, A review of tectonic, elastic and visco-elastic models exploring the deformation patterns throughout the eruption of Soufrière Hills volcano on Montserrat, West Indies. *J. Volcanol. Geotherm. Res.* **425**, 107518 (2022).

35. S. R. Young, P. W. Francis, J. Barclay, T. J. Casadevall, C. A. Gardner, B. Darroux, M. A. Davies, P. Delmelle, G. E. Norton, A. J. H. Maciejewski, C. M. M. Oppenheimer, J. Stix, I.

M. Watson, Monitoring SO_2 emission at the Soufriere Hills Volcano: Implications for changes in eruptive conditions. *Geophys. Res. Lett.* **25**, 3681–3684 (1998).

36. M. Edmonds, R. A. Herd, B. Galle, C. M. Oppenheimer, Automated, high time-resolution measurements of SO_2 flux at Soufrière Hills Volcano, Montserrat. *Bull. Volcanol.* **65**, 578–586 (2003).

37. Scientific Advisory Committee (SAC), "Assessment of the hazards and risks associated with the Soufriere Hills volcano, Montserrat," *22nd Report of the Scientific Advisory Committee on Montserrat Volcanic Activity* (Scientific Advisory Committee on Montserrat Volcanic Activity, 2018).

38. T. Christopher, M. Edmonds, M. C. S. Humphreys, R. A. Herd, Volcanic gas emissions from Soufrière Hills Volcano, Montserrat 1995–2009, with implications for mafic magma supply and degassing. *Geophys. Res. Lett.* **37**, L00E04 (2010).

39. H. Shinohara, Excess degassing from volcanoes and its role on eruptive and intrusive activity. *Rev. Geophys.* **46**, RG4005 (2008).

40. P. J. Wallace, Volcanic SO_2 emissions and the abundance and distribution of exsolved gas in magma bodies. *J. Volcanol. Geotherm. Res.* **108**, 85–106 (2001).

41. M. Edmonds, D. Pyle, C. Oppenheimer, HCl emissions at Soufrière Hills Volcano, Montserrat, West Indies, during a second phase of dome building: November 1999 to October 2000. *Bull. Volcanol.* **64**, 21–30 (2002).

42. C. Oppenheimer, M. Edmonds, P. Francis, M. Burton, Variation in HCl/SO_2 gas ratios observed by Fourier transform spectroscopy at Soufrière Hills Volcano, Montserrat. *Geol. Soc. Lond. Mem.* **21**, 621–639 (2002).

43. M. Edmonds, A. Aiuppa, M. Humphreys, R. Moretti, G. Giudice, R. S. Martin, R. A. Herd, T. Christopher, Excess volatiles supplied by mingling of mafic magma at an andesite arc volcano. *Geochem. Geophys. Geosyst.* **11**, Q04005 (2010).

44. B. Esse, M. Burton, M. Varnam, R. Kazahaya, G. Salerno, iFit: A simple method for measuring volcanic SO_2 without a measured Fraunhofer reference spectrum. *J. Volcanol. Geotherm. Res.* **402**, 107000 (2020).

45. G. Chiodini, R. Cioni, A. Frullani, M. Guidi, L. Marini, F. Prati, B. Raco, Fluid geochemistry of Montserrat Island, West Indies. *Bull. Volcanol.* **58**, 380–392 (1996).

46. C. Oppenheimer, P. R. Kyle, Probing the magma plumbing of Erebus volcano, Antarctica, by open-path FTIR spectroscopy of gas emissions. *J. Volcanol. Geotherm. Res.* **177**, 743–754 (2008).

47. M. Edmonds, D. Pyle, C. Oppenheimer, A model for degassing at the Soufrière Hills Volcano, Montserrat, West Indies, based on geochemical data. *Earth Planet. Sci. Lett.* **186**, 159–173 (2001).

48. A. Aiuppa, T. P. Fischer, T. Plank, P. Bani, CO_2 flux emissions from the Earth's most actively degassing volcanoes, 2005–2015. *Sci. Rep.* **9**, 5442 (2019).

49. M. R. Burton, G. M. Sawyer, D. Granieri, Deep carbon emissions from volcanoes. *Rev. Mineral. Geochem.* **75**, 323–354 (2013).

50. T. P. Fischer, S. Arellano, S. Carn, A. Aiuppa, B. Galle, P. Allard, T. Lopez, H. Shinohara, P. Kelly, C. Werner, C. Cardellini, G. Chiodini, The emissions of CO_2 and other volatiles from the world's subaerial volcanoes. *Sci. Rep.* **9**, 18716 (2019).

51. C. Werner, T. P. Fischer, A. Aiuppa, M. Edmonds, C. Cardellini, S. Carn, G. Chiodini, E. Cottrell, M. Burton, H. Shinohara, P. Allard, "Carbon dioxide emissions from subaerial volcanic regions: Two decades in review" in *Deep Carbon: Past to Present*, B. N. Orcutt, I. Daniel, R. Dasgupta, Eds. (Cambridge Univ. Press, 2019), pp. 188–236.

52. P. Allard, J. Carbonnelle, D. Dajlevic, J. L. Bronec, P. Morel, M. C. Robe, J. M. Maurenas, R. Faivre-Pierret, D. Martin, J. C. Sabroux, P. Zettwoog, Eruptive and diffuse emissions of CO_2 from Mount Etna. *Nature* **351**, 387–391 (1991).

53. E. J. Liu, K. Wood, E. Mason, M. Edmonds, A. Aiuppa, G. Giudice, M. Bitetto, V. Francofonte, S. Burrow, T. Richardson, M. Watson, T. D. Pering, T. C. Wilkes, A. J. S. McGonigle, G. Velasquez, C. Melgarejo, C. Bucarey, Dynamics of outgassing and plume transport revealed by proximal unmanned aerial system (UAS) measurements at Volcán Villarrica, Chile. *Geochem. Geophys. Geosyst.* **20**, 730–750 (2019).

54. G. Tamburello, Ratiocalc: Software for processing data from multicomponent volcanic gas analyzers. *Comput. Geosci.* **82**, 63–67 (2015).

55. K. Wood, E. J. Liu, A. Aiuppa, G. Gludice, M. Bitetto, T. Richardson, “A deconvolution-based sensor response correction for volcanic gas measurements”, Poster F41, Deep Carbon 2019.

56. E. J. Liu, A. Aiuppa, A. Alan, S. Arellano, M. Bitetto, N. Bobrowski, S. Carn, R. Clarke, E. Corrales, J. M. de Moor, J. A. Diaz, M. Edmonds, T. P. Fischer, J. Freer, G. M. Fricke, B. Galle, G. Gerdes, G. Giudice, A. Gutmann, C. Hayer, I. Itikarai, J. Jones, E. Mason, B. T. McCormick Kilbride, K. Mulina, S. Nowicki, K. Rahilly, T. Richardson, J. Rüdiger, C. I. Schipper, I. M. Watson, K. Wood, Aerial strategies advance volcanic gas measurements at inaccessible, strongly degassing volcanoes. *Sci. Adv.* **6**, eabb9103 (2020).

57. T. D. Pering, E. J. Liu, K. Wood, T. C. Wilkes, A. Aiuppa, G. Tamburello, M. Bitetto, T. Richardson, A. J. S. McGonigle, Combined ground and aerial measurements resolve vent-specific gas fluxes from a multi-vent volcano. *Nat. Commun.* **11**, 3039 (2020).

58. J. Rufus, G. Stark, A. P. Thorne, J. C. Pickering, R. J. Blackwell-Whitehead, D. Blackie, P. L. Smith, High-resolution photoabsorption cross-section measurements of SO_2 at 160 K between 199 and 220 nm. *J. Geophys. Res. Planets.* **114**, E06003 (2009).

59. V. Gorshelev, A. Serdyuchenko, M. Weber, W. Chehade, J. P. Burrows, High spectral resolution ozone absorption cross-sections—Part 1: Measurements, data analysis and comparison with previous measurements around 293 K. *Atmos. Meas. Tech.* **7**, 609–624 (2014).

60. T. Danckaert, C. Fayt, M. Van Roozendael, QDOAS Software user manual. Technical Report Royal Belgian Institute for Space Aeronomy (2017); https://uv-vis.aeronomie.be/software/QDOAS/.

61. K. Chance, R. L. Kurucz, An improved high-resolution solar reference spectrum for earth's atmosphere measurements in the ultraviolet, visible, and near infrared. *J. Quant. Spectrosc. Radiat. Transfer.* **111**, 1289–1295 (2010).

62. I. E. Gordon, L. S. Rothman, R. J. Hargreaves, R. Hashemi, E. V. Karlovets, F. M. Skinner, E. K. Conway, C. Hill, R. V. Kochanov, Y. Tan, P. Wcisło, A. A. Finenko, K. Nelson, P. F. Bernath, M. Birk, V. Boudon, A. Campargue, K. V. Chance, A. Coustenis, B. J. Drouin, J.-M. Flaud, R. R. Gamache, J. T. Hodges, D. Jacquemart, E. J. Mlawer, A. V. Nikitin, V. I. Perevalov, M. Rotger, J. Tennyson, G. C. Toon, H. Tran, V. G. Tyuterev, E. M. Adkins, A. Baker, A. Barbe, E. Canè, A. G. Császár, A. Dudaryonok, O. Egorov, A. J. Fleisher, H. Fleurbaey, A. Foltynowicz, T. Furtenbacher, J. J. Harrison, J.-M. Hartmann, V.-M. Horneman, X. Huang, T. Karman, J. Karns, S. Kassi, I. Kleiner, V. Kofman, F. Kwabia-Tchana, N. N. Lavrentieva, T. J. Lee, D. A. Long, A. A. Lukashevskaya, O. M. Lyulin, V. Y. Makhnev, W. Matt, S. T. Massie, M. Melosso, S. N. Mikhailenko, D. Mondelain, H. S. P. Müller, O. V. Naumenko, A. Perrin, O. L. Polyansky, E. Raddaoui, P. L. Raston, Z. D. Reed, M. Rey, C. Richard, R. Tóbiás, I. Sadiek, D. W. Schwenke, E. Starikova, K. Sung, F. Tamassia, S. A. Tashkun, J. Vander Auwera, I. A. Vasilenko, A. A. Vigasin, G. L. Villanueva, B. Vispoel, G. Wagner, A. Yachmenev, S. N. Yurchenko, The HITRAN2020 molecular spectroscopic database. *J. Quant. Spectrosc. Radiat. Transfer.* **277**, 107949 (2022).

63. A. Chiarugi, S. Viciani, F. D'Amato, M. Burton, Diode laser-based gas analyser for the simultaneous measurement of CO_2 and HF in volcanic plumes. *Atmos. Meas. Tech.* **11**, 329–339 (2018).

64. J. A. Coxon, P. G. Hajigeorgiou, Improved direct potential fit analyses for the ground electronic states of the hydrogen halides: HF/DF/TF, HCl/DCl/TCl, HBr/DBr/TBr and HI/DI/TI. *J. Quant. Spectrosc. Radiat. Transfer.* **151**, 133–154 (2015).

65. K. Bogumil, J. Orphal, T. Homann, S. Voigt, P. Spietz, O. C. Fleischmann, A. Vogel, M. Hartmann, H. Kromminga, H. Bovensmann, J. Frerick, J. P. Burrows, Measurements of

molecular absorption spectra with the SCIAMACHY pre-flight model: Instrument characterization and reference data for atmospheric remote-sensing in the 230–2380 nm region. *J. Photochem. Photobiol. A Chem.* **157**, 167–184 (2003).
